# A Supramolecular Nanoscavengers: Based “Cargo‐Exchange” Breaking Cholesterol Metabolic Dysregulation for Glioblastoma Therapy

**DOI:** 10.1002/advs.202519690

**Published:** 2025-11-20

**Authors:** Zonghua Tian, Yun Chen, Jingyi Zhou, Shilin Zhang, Hongrui Fan, Xuwen Li, Tao Sun, Chen Jiang

**Affiliations:** ^1^ Department of Pharmaceutics School of Pharmaceutical Sciences Fudan University Key Laboratory of Smart Drug Delivery Ministry of Education State Key Laboratory of Brain Function and Disorders Shanghai 201203 China

**Keywords:** blood‐brain barrier, cholesterol metabolic dysregulation, glioblastoma, host‐guest recognition, immunosuppressive tumor microenvironment, supramolecule

## Abstract

Dysregulated cholesterol metabolism, driven by a complex array of mechanisms, is a defining characteristic of glioblastoma (GBM) and plays a crucial role in the development of resistance to therapeutic interventions. In this study, supramolecular nanoscavengers are engineered by harnessing the intrinsic binding affinity between endogenous small‐molecule cholesterol and β‐cyclodextrin (β‐CD). This host‐guest interaction enabled both targeted drug release and cholesterol clearance through competitive displacement within the cholesterol‐rich microenvironment of GBM. Consequently, tumor‐associated cholesterol dysregulation is effectively disrupted, resulting in significant inhibition of cellular proliferation and invasion. Furthermore, the supramolecular nanoscavengers interfered with the metabolic crosstalk between tumor cells and immune cells involving cholesterol, thereby remodeling the immunosuppressive tumor microenvironment and demonstrating substantial therapeutic efficacy in vivo. Additionally, the dynamic and reversible binding between β‐CD and various guest molecules facilitated efficient delivery of the supramolecular host to the central nervous system (CNS). Collectively, these findings presented a promising strategy for targeting cholesterol as an intervention in the treatment of GBM as well as other CNS disorders associated with altered cholesterol metabolism.

## Graphical abstract

Dysregulated cholesterol metabolism, driven by a complex array of mechanisms, is a defining characteristic of glioblastoma (GBM). In this study, supramolecular nanoscavengers are engineered by harnessing the intrinsic binding affinity between endogenous small‐molecule cholesterol and β‐cyclodextrin (β‐CD). This host‐guest interaction enabled both targeted drug release and cholesterol clearance through competitive displacement within the cholesterol‐rich microenvironment of GBM.

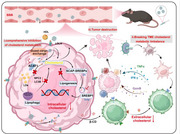

## 1 Introduction

Glioblastoma (GBM) is the most prevalent primary malignant tumor of the central nervous system (CNS), distinguished by its infiltrative growth and significant cellular heterogeneity.^[^ ^1^, ^2^ ^]^ The current standard treatment employs a multimodal approach that integrates maximal safe surgical resection with radiotherapy and chemotherapy. Nevertheless, the efficacy of these therapeutic modalities is constrained by several factors, including intricate signaling pathways, intratumoral genetic diversity, the blood‐brain barrier (BBB), and an immunosuppressive tumor microenvironment.^[^ ^3^, ^4^, ^5^ ^]^ These challenges underscore the pressing need to develop precise and effective targeted treatment strategies for GBM.

In the processes of tumorigenesis and tumor progression, cancer cells engage in continuous metabolic adaptation to optimally respond to their changing environment. Among these adaptations, tumor cells reprogram lipid metabolism to acquire essential energy sources, biofilm components, and signaling molecules within the tumor microenvironment that are critical for proliferation, survival, invasion, and metastasis.^[^ ^6^, ^7^, ^8^ ^]^ Notably, GBM exhibits a prominent characteristic of lipid metabolism reprogramming. It is important to highlight that cholesterol—a crucial lipid molecule for maintaining the structure and function of the CNS‐is subject to limited or negligible transport from peripheral circulation into the CNS due to stringent restrictions imposed by the BBB. As a result, the cholesterol required by the brain predominantly arises from endogenous synthesis.^[^ ^9^, ^10^ ^]^ Under normal physiological conditions, cholesterol concentration in the brain is maintained within a specific range known as brain cholesterol homeostasis; this balance is vital for sustaining normal CNS function and development.^[^ ^11^, ^12^, ^13^ ^]^ However, cholesterol homeostasis within GBM cells is dynamically regulated by multiple factors including uptake, synthesis, and efflux mechanisms.^[^ ^14^ ^]^ The metabolism of cholesterol in GBM cells operates with high autonomy and distinctiveness. This suggests that cholesterol metabolism in GBM presents manipulable local vulnerabilities—offering promising strategies for targeted treatment through modulation of cholesterol metabolic pathways.

Research findings indicated that within the GBM microenvironment, tumor cells exhibit a pronounced dependence on the uptake and storage of exogenous cholesterol to satisfy their rapid proliferation demands.^[^ ^15^ ^]^ This reliance leads to an increased distribution of cholesterol within the cell membrane, particularly in lipid raft domains, consequently, which facilitates the formation of invasive pseudopodia, thereby enhancing the invasive capacity of tumor cells.^[^ ^16^ ^]^ Simultaneously, when exogenous cholesterol uptake is insufficient, it triggers the activation of sterol regulatory element‐binding proteins (SREBPs), which are critical transcription factors regulating lipid synthesis. This activation initiates de novo synthesis pathways for cholesterol and activates protective lipid autophagy signaling pathways that hydrolyze lipid droplets to sustain cholesterol supply.^[^ ^17^, ^18^ ^]^ Furthermore, an expanding body of research has demonstrated that beyond promoting tumor development, reprogramming of cholesterol metabolism also alters the tumor microenvironment by influencing immune cell functions and infiltration. The “cholesterol‐rich” phenomenon induced by tumors accelerates the establishment of an immunosuppressive microenvironment. For instance, it induces functional exhaustion in cytotoxic CD8^+^ T cells, diminishing their ability to eliminate tumor cells. It also promotes infiltration by macrophages with inhibitory phenotypes and regulatory T cells, thus creating a paradoxical scenario regarding cholesterol metabolism that can resist treatment.^[^ ^19^, ^20^, ^21^ ^]^ Consequently, multi‐faceted regulation and disruption of the intricate network surrounding contradictions in cholesterol metabolism between tumor cells and immune cells within the GBM microenvironment represent effective strategies for inhibiting tumor cell proliferation and alleviating immunosuppression. However, due to the stringent nature of the BBB, precisely targeting therapeutic drugs to tumor lesion sites and effectively accumulating these drugs at those locations to achieve a therapeutically relevant concentration remains a significant challenge.^[^ ^22^ ^]^

The active‐targeting nanodelivery system platform has demonstrated remarkable potential in the realm of brain drug delivery, attributed to its adjustable dimensions, surface charge characteristics, and capacity for diverse ligand modifications.^[^ ^23^, ^24^ ^]^ Recently, supramolecular nanomaterials have garnered significant attention as they serve dual roles as both drug carriers and therapeutic agents. These materials offer several advantages, including high stability, robust inclusion capabilities, and adaptability to modification. They primarily form specific complexes through “weak interactions” between guest and host molecules—such as electrostatic forces, *π*–*π* stacking interactions, and hydrogen bonding.^[^ ^25^, ^26^, ^27^ ^]^ β‐Cyclodextrin (β‐CD), a drug solubilizer recognized as safe by the Food and Drug Administration (FDA), serves as an exemplary foundation for the development of supramolecular drug delivery systems. As a type of nanocarrier, it demonstrates remarkable potential for clinical translation. For instance, the renal‐clearable zwitterionic CD nanostructures developed by Jae‐Young Lee could effectively deliver therapeutic agents to tumor sites.^[^ ^28^ ^]^ In addition to its role as a carrier, β‐CD demonstrates notable therapeutic potential. The cavity of β‐CD exhibits a high degree of structural compatibility with the steroid nucleus of cholesterol, a crucial lipid molecule that is widely distributed throughout biological systems. Consequently, it can form a stable complex with cholesterol, thereby facilitating its clearance from the body. This effective cholesterol‐clearing capability has been convincingly validated in experimental models of atherosclerosis. Furthermore, clinical trials are currently underway for Niemann‐Pick disease type C1, a disorder characterized by impaired cholesterol transport.^[^ ^29^, ^30^ ^]^ However, several challenges associated with β‐CD, including limited water solubility, inability to effectively penetrate the BBB, intolerance to intrathecal injection procedures, non‐specific binding to circulating cholesterol leading to hemolysis issues, and non‐specific distribution patterns have severely limited its application in CNS diseases, especially those related to cholesterol metabolism disorders.^[^ ^31^, ^32^ ^]^ Therefore, it is imperative to modify the structure of β‐CD through chemical modification to safely, effectively and precisely transport it to the CNS to regulate cholesterol metabolism disorders and treat GBM.

**Scheme 1:**
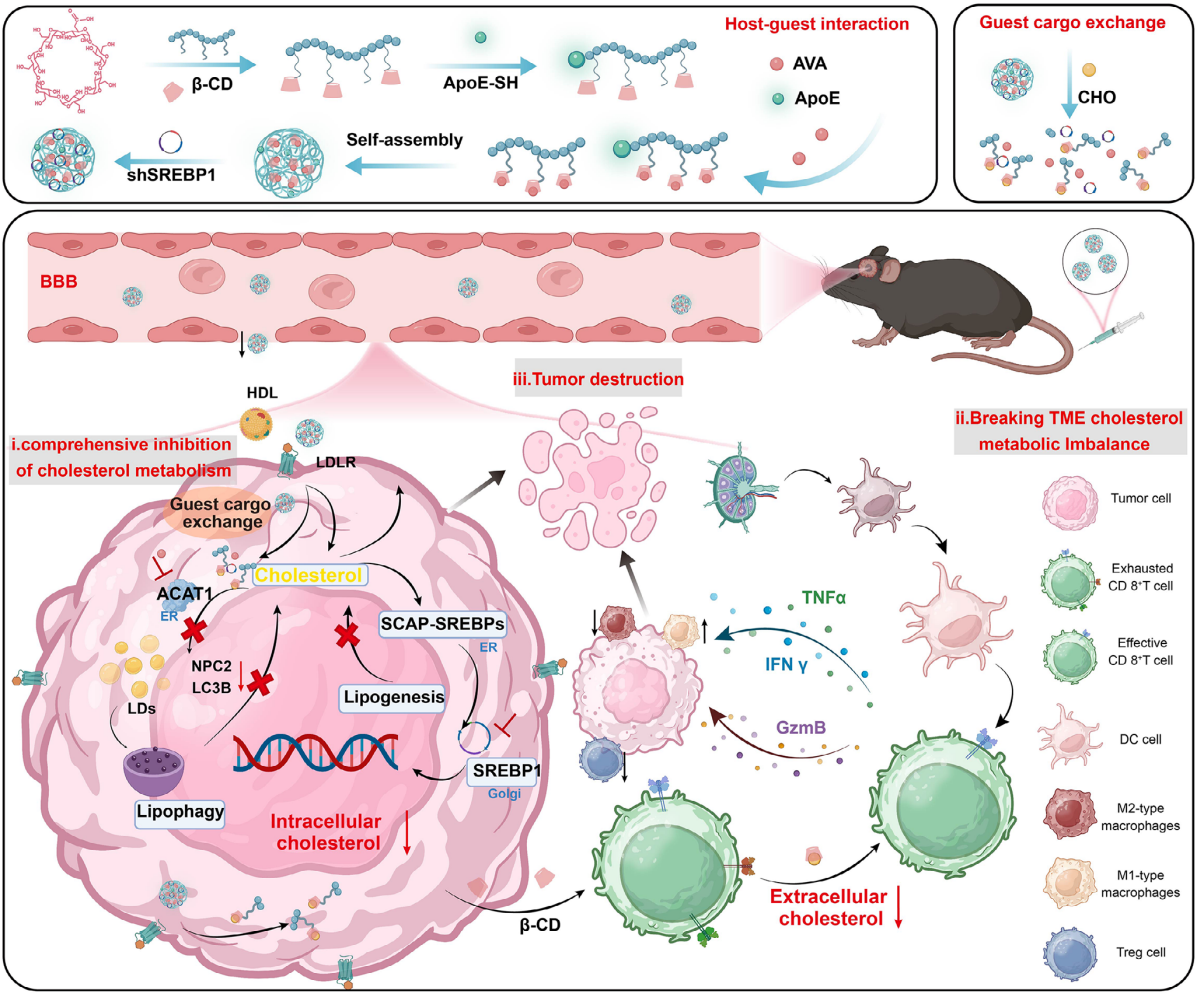
Schematic illustration of supramolecular nanoscavengers extensively modulated cholesterol imbalance in glioblastoma based “host‐exchange” mechanism, concurrently remodeling the immunosuppressive tumor microenvironment. Graphic created with BioRender.com.

In light of the cholesterol metabolic reprogramming and the stringent BBB characteristics exhibited by GBM, this research exploited the natural affinity between β‐CD and cholesterol to construct a supramolecular nanoscavengers grounded on the cargo‐exchange mechanism (**Scheme** **1**). Moreover, this supramolecular nanoscavengers was modified with ApoE polypeptide, endowing it with the ability to bind to the LDLR receptors that are highly expressed in the BBB and tumor cells. In the cholesterol‐enriched tumor microenvironment, given that the natural affinity between cholesterol and β‐CD surpasses that between β‐CD and avasimibe (AVA), through host‐guest recognition‐exchange interactions, the drug molecule AVA was liberated to fulfill its function of inhibiting the cholesterol esterification process. By way of negative feedback regulation, the uptake of cholesterol also diminished. Simultaneously, the released shSREBP1 downregulated the expression of SREBP1, thereby impeding the activation of the tumor's intrinsic cholesterol synthesis pathway and suppressing the emergence of protective lipophagy. In this manner, the cholesterol supply to tumor cells was comprehensively obstructed, and their invasion and proliferation was constrained. Simultaneously, β‐CD performed the dual functions of a carrier‐scavenger. Within the tumor microenvironment, β‐CD further bound to cholesterol, thereby forming a cholesterol‐CD complex. This binding behavior was instrumental in scavenging excess cholesterol, which in turn contributed to reducing CD8^+^ T cell exhaustion, inhibiting the infiltration of inhibitory macrophages and regulatory T cells, and finally remodeling the immunosuppressive microenvironment. Ultimately, through multi‐angle regulation of cholesterol metabolism and breaking the intricate network of contradictions in cholesterol metabolism between tumor cells and immune cells, offering a novel perspective and a promising approach for the treatment of GBM.

## 2 Results and Discussion

### 2.1 Verification of the Reprogramming Characteristics of Cholesterol Metabolism in GBM

Cholesterol is a ubiquitous and vital component within cells, serving a critical function in preserving cellular viability and functionality. Cholesterol metabolism can generate a diverse array of bioactive intermediates through the activation of multiple signaling pathways. These intermediates not only regulate various signaling cascades but also play crucial roles in modulating numerous cellular processes, including cell growth, proliferation, differentiation, survival, apoptosis, inflammation, motility, and the maintenance of cell membrane homeostasis.^[^ ^33^, ^34^ ^]^ Among the various organs, the brain stands out as the one with the highest cholesterol content, comprising ≈25% of the total body cholesterol. However, due to the presence of the BBB, the cholesterol required by the brain primarily derives from endogenous synthesis.^[^ ^35^ ^]^ In comparison to normal cells, the dysregulation of cholesterol metabolism represents one of the defining characteristics of malignant GBM. Furthermore, this process is dynamically regulated by a variety of mechanisms, including uptake, synthesis, storage, and efflux. Research findings indicate that GBM cells predominantly utilize cholesterol through the uptake of exogenous cholesterol via the highly expressed low‐density lipoprotein receptor (LDLR).^[^ ^36^ ^]^ Within these cells, acyl‐coenzyme A: cholesterol acyltransferase 1 (ACAT1) functions as the sole enzyme responsible for esterifying free cholesterol into lipid droplets for storage. This process is crucial in regulating cholesterol metabolism by maintaining a dynamic equilibrium between free and esterified forms of cholesterol within the cellular environment.^[^ ^37^ ^]^ Moreover, stringent regulation of a complex protein network centered around sterol‐regulatory element binding proteins (SREBPs) and liver X receptors (LXRs) plays an essential role in mediating processes such as cholesterol influx, efflux, synthesis, metabolism, and esterification.^[^ ^38^, ^39^, ^40^ ^]^

To evaluate the characteristics of cholesterol reprogramming within the GBM microenvironment, we investigated the expression levels of ACAT1 and SREBPs by using immunofluorescence staining techniques following the successful establishment of an intracranial in situ mouse model and integrating data from The Cancer Genome Atlas (TCGA) database. Following the in situ injection of tumor cells, the intracranial in situ GBM model was successfully established, as confirmed by HE staining (Figure S1a, Supporting Information). Bioinformatics analysis of the database revealed that, in comparison to normal tissues, tumor tissues exhibited significantly elevated expression levels of ACAT1, as well as lipid metabolism‐related proteins such as SREBP1, LDLR, and SQLE (Figure S1b, Supporting Information). Immunofluorescence results indicated that, relative to normal mouse brain tissue, tumor tissue demonstrated markedly higher expression of ACAT1 esterase and SREBP1, along with a significantly greater distribution of free cholesterol and lipid droplets (Figure S1c,d, Supporting Information). In addition, consistent results were also observed in the validation of GBM patient samples (Figure S2, Supporting Information), which aligned with the characteristics of cholesterol metabolic reprogramming in GBM as documented in the existing literature.^[^ ^17^ ^]^ Overall, the aforementioned research findings provided strong support for the continued advancement of this study.

### 2.2 Preparation and Characterization of “Guest Cargo‐Exchange” Supramolecular Nanoscavengers

β‐CD, an excipient or formulation agent approved by the FDA, possesses a unique structure characterized by its hydrophilic exterior and hydrophobic inner cavity. This property enables it to function as a host molecule, selectively encapsulating guest molecules. For drugs with low solubility, encapsulation within the cavity of cyclodextrin presents a viable strategy, which not only enhances the solubility of these drugs but also has the potential to improve their bioavailability.^[^ ^41^ ^]^ However, natural β‐CD exhibits certain limitations in drug delivery applications. Specifically, its poor water solubility and propensity to induce hemolysis during parenteral administration restrict its utility. Through chemical modification techniques such as alkylation, hydroxy‐alkylation, or esterification of the free hydroxyl groups present in the structure, it is possible to synthesize cyclodextrin derivatives that exhibit enhanced water solubility, amorphous characteristics, high physical stability, and low physiological toxicity, which significantly improve their performance and broaden their range of applications.^[^ ^42^, ^43^, ^44^ ^]^ Here, on the premise of not affecting the cavity of β‐CD, we carried out hydrophilic chain modification on the hydroxyl group at the C6 position. Specifically, a positively charged polyline‐arginine hydrophilic chain segment was mainly introduced. On the one hand, this modification increased the hydrophilicity; on the other hand, the positively charged groups could interact with shSREBP1 through positive‐negative electrostatic interactions, thereby achieving the co‐delivery of hydrophobic drugs and gene drugs.

Following the synthetic route (Figure S3, Supporting Information), we successfully synthesized brush‐like polyamine acid β‐CD‐based polymers and ApoE‐modified polyamine acid β‐CD‐based polymers. The products and intermediates were characterized by ^1^H NMR, confirming the successful synthesis of the brush‐like polyamine acid β‐CD‐based polymers (Figures S4–S13, Supporting Information), which were deemed suitable for subsequent investigations. Furthermore, we examined the impact of these polymers on cellular cholesterol levels. The experimental results indicated that, in comparison to the parent β‐CD, both brush‐like polyamine acid β‐CD‐based polymers retained the capacity to clear cholesterol (Figure S14, Supporting Information). This finding suggested that the modification with hydrophilic chains preserves the integrity of the hydrophobic cavity of β‐CD, which was crucial for the development of nanoscavengers. Building upon the successful synthesis of brush‐like polyamine acid β‐CD‐based polymers, we first assessed the feasibility of these polymers forming a nano‐delivery system. Utilizing pyrene as a hydrophobic probe, we determined the critical aggregation concentration (Figure S15, Supporting Information). The experimental results demonstrated that as the polymer concentration approached a critical threshold, the fluorescence intensity of pyrene increased, while the β‐CD group exhibited no significant change in fluorescence intensity. This observation implied that the binding of β‐CD to the polyamine acid backbone facilitated the incorporation of hydrophobic molecules into the hydrophobic cavity, thereby enabling the formation of a nano‐delivery system through host‐guest recognition and self‐assembly.

**Figure 1:**
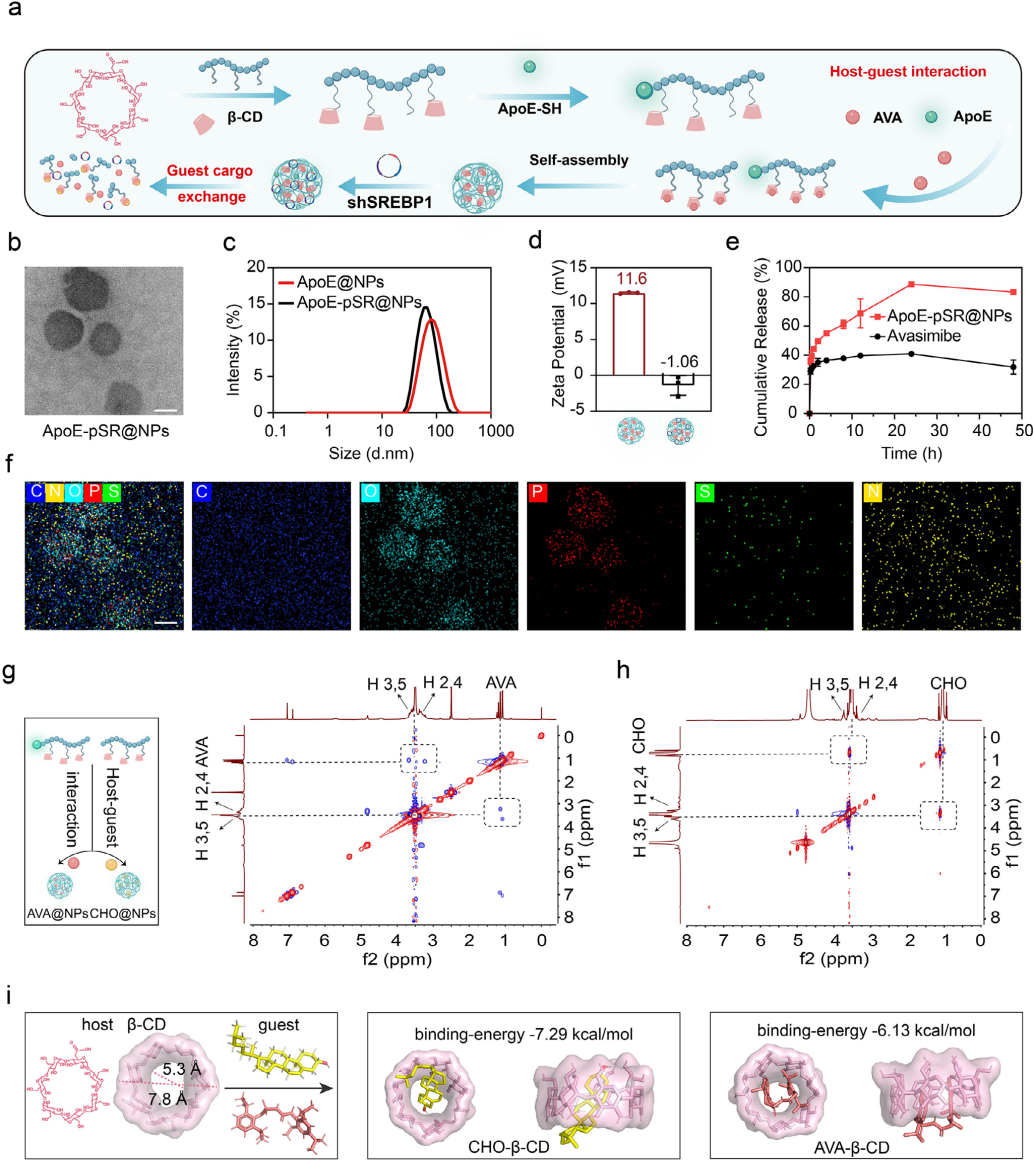
Preparation and characterization of supramolecular nanoscavengers. a) Schematic diagram of the preparation of the supramolecular nanoscavengers delivery system. b) TEM morphology image of supramolecular nanoscavengers, scale bars, 100 nm. c,d) Particle size and potential of the supramolecular nanoscavengers including and excluding plasmid shSREBP1 (*n* = 3). e) In vitro release of avasimibe from the supramolecular nanoscavengers (*n* = 3). f) Elemental energy spectrum analysis of the supramolecular nanoscavengers by TEM, scale bars, 100 nm. g,h) ROESY spectra of complexes formed with different guest molecules (avasimibe or cholesterol). i) Docking results of host β‐CD with different guest (cholesterol/avasimibe) molecules.

To co‐deliver the gene drug shSREBP1 and the ACAT1 inhibitor AVA to tumor tissues, we first used the nano‐precipitation method to form a nano‐core through host‐guest recognition between the hydrophobic drug AVA and the polymer core β‐CD, which have been optimized with respect to particle size, drug loading, and encapsulation efficiency. Subsequently, shRNA was adsorbed and condensed through the interaction between positively and negatively charged entities (**Figure** **1**a). Among the aforementioned, the optimal binding ratio of the nano‐core to the gene shSREBP1 was determined through agarose gel electrophoresis. The results indicated that a mass ratio of 50:1 between the nano‐core and gene shSREBP1 facilitated successful complex formation (Figure S16, Supporting Information), thereby enhancing their co‐delivery potential. Subsequently, using dynamic light scattering (DLS) and TEM to investigate the particle size, potential, stability, and morphology of the integrated “cargo‐exchange” supramolecular nanoscavengers, results showed that the successfully constructed nanoscavengers exhibited a spherical and uniform distribution (Figure 1b). The particle size of the supramolecular nanoscavengers was ≈100 nm, and the drug loading of AVA in the supramolecular nanoscavengers was determined to be ≈7% by HPLC, with an encapsulation efficiency of ≈67%. The addition of the gene reduced the zeta potential to ≈−1 mV, which was close to neutral (Figure 1c,d). In addition, the in vitro release behavior of the hydrophobic drug AVA and shSREBP1 were investigated. The experimental results showed that the release behavior of AVA was significantly improved after nano‐treatment. Simultaneously, shSREBP1 was also released in accordance with this process, which provided the possibility for AVA and shSREBP1 to exert their effects (Figure 1e; Figure S17, Supporting Information). Furthermore, we analyzed all the elements contained in the “cargo‐exchange” supramolecular nanoscavengers by transmission electron microscopy‐surface element energy spectrum analysis. The results revealed that the characteristic element S of AVA and the unique element P of the gene were evenly distributed in the overall contour of the nanoparticles, which fully revealed that the nano‐core and the gene drug formed an integrated supramolecular nanoscavengers through electrostatic interactions (Figure 1f). Equally significant is the fact that the therapeutic efficacy of the poly‐β‐CD‐based nano‐delivery system is intricately linked to its stability and biocompatibility with blood. We conducted a short‐term stability assessment of the successfully synthesized supramolecular nanoscavengers. The experimental results demonstrated both key supramolecular nanoscavengers' excellent stability over a 7‐day period under storage and simulated physiological environmental conditions (Figure S18, Supporting Information) with a low risk of hemolysis (Figure S19, Supporting Information), suggesting that hydrophilic modification and pre‐occupation of the β‐CD cavity contribute to enhanced safety in vivo transport of β‐CD, which provided a basis for the systemic administration of β‐CD. Furthermore, using a similar approach, we developed supramolecular nanoscavengers that encapsulated the DID fluorescent probe. These nanoscavengers demonstrate particle sizes and zeta potentials comparable to those of AVA‐loaded nanoformulations (Figure S20, Supporting Information).

Through the above series of characterizations, we demonstrated the successful preparation of a nano‐delivery system formed by AVA as a hydrophobic guest molecule and β‐CD through host‐guest recognition. Then, we further investigated the possibility of the supramolecular nanoscavengers achieving cargo exchange, which is crucial for the therapeutic effect of the delivery system. First, we formed two types of nano‐delivery systems using two guest molecules, AVA and cholesterol, respectively, with brush‐like polyamine acid β‐CD‐based polymers through host‐guest recognition. We then analyzed the hydrogen‐hydrogen interactions using 2D HNMR ROESY. The spectra showed that both guest molecules could interact with the polymer parent β‐CD, indicating that both guest molecules could enter the β‐CD cavity. The complexes formed by the two served as hydrophobic cores to further drive the formation of the nano‐delivery system (Figure 1g,h). Simultaneously, we used molecular docking software to simulate the binding of the two guest molecules and the β‐CD parent and calculated the binding energy of the complexes. The results revealed that the binding energy of the complex formed by cholesterol and β‐CD was significantly greater than that of the complex formed by AVA and β‐CD (Table S1, Supporting Information), which fully indicated that the structures of both guest molecules can match the cavity of β‐CD to a certain extent, and the structural matching degree of cholesterol was superior to that of AVA. This further suggested that when AVA pre‐occupied the β‐CD cavity, the preferential matching ability of cholesterol can promote the “cargo exchange” phenomenon, which was beneficial for the release of AVA and the reduction of cholesterol concentration. At the same time, β‐CD fully played a dual role as a delivery carrier and cholesterol clearance agent, which was a very important basis for the “cargo‐exchange” supramolecular nanoscavengers we constructed to regulate cholesterol metabolism reprogramming for the treating of GBM.

### 2.3 Screening of ApoE Targeting Functional Element Modification Ratio, Uptake Pathway, and Intracellular Fate of Supramolecular Nanoscavengers

**Figure 2:**
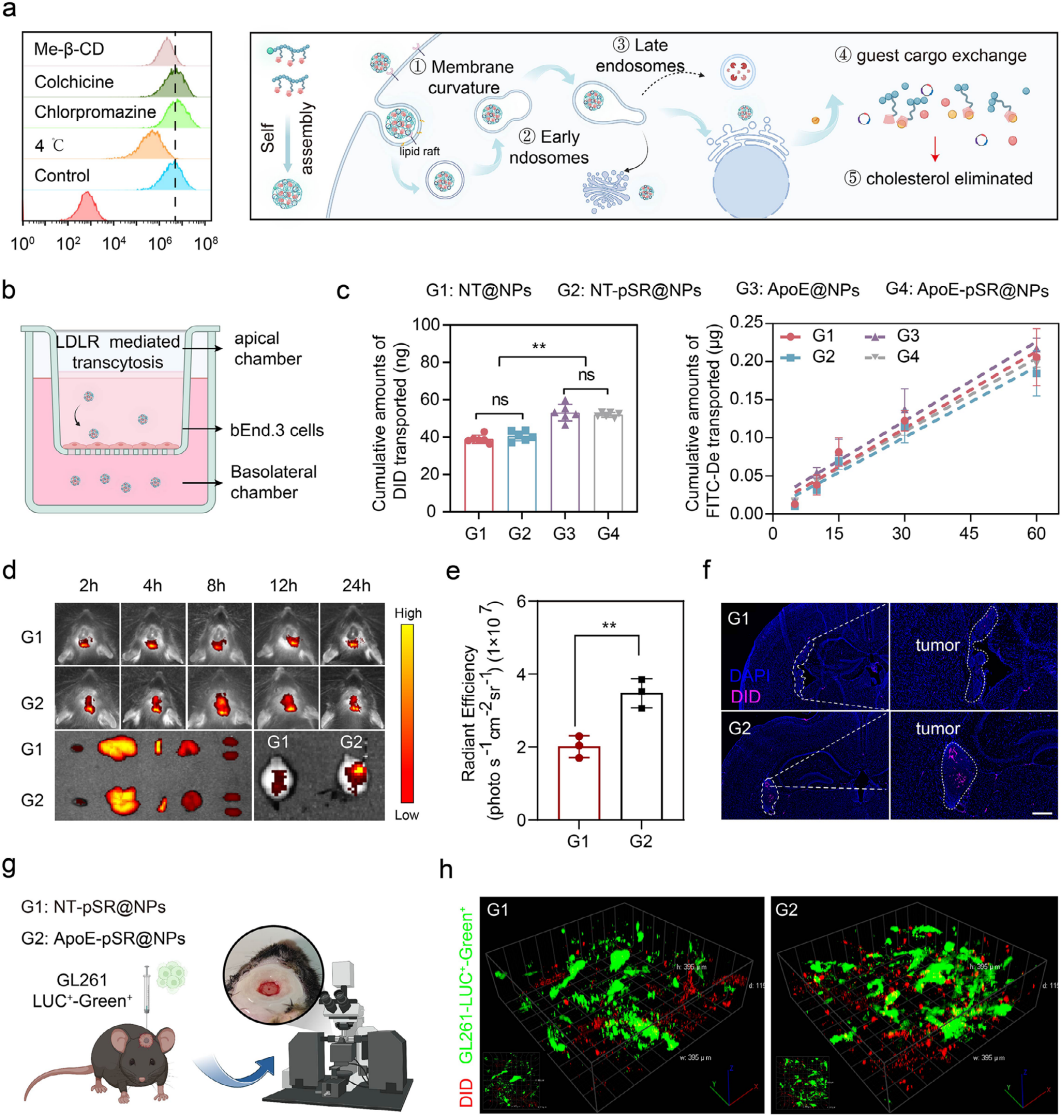
Experimental results of supramolecular nanoscavengers targeting at the cellular and animal levels. a) the uptake pathways and schematic diagram of supramolecular nanoscavengers (*n* = 3). b,c) In vitro BBB model penetration experimental results of supramolecular nanoscavengers (*n* = 3). d–f) In vivo tumor targeting experimental results of supramolecular nanoscavengers, purple represented the supramolecular nanoscavengers were labeled with the DID probe, blue represents the cell nucleus, scale bars, 200 µm. g,h) Schematic diagram and results of two‐photon imaging of the supramolecular nanoscavengers targeting tumor sites in vivo, green signified the GBM organization, red denoted the nanoscavengers labeled with the DID probe. The data in (c and e) were shown in analytic plots as mean ± s.d., and ordinary one‐way ANOVA was used (*p < 0.05, **p < 0.01, ***p < 0.001, and ****p < 0.0001*)*.

ApoE is the primary cholesterol carrier protein in the CNS, facilitating cholesterol transport through binding to the LDLR receptor on cell surfaces, which is highly expressed on GBM cells and the BBB.^[^ ^45^ ^]^ The modification of supramolecular nanoscavengers with ApoE enables active targeting of the lesion site by the nano‐delivery system, allowing for accumulation and laying the groundwork for drug efficacy. Experimental results indicated that at the cellular level, a 30% ApoE modification ratio yielded the highest cellular uptake (Figure S21, Supporting Information). Thus, we selected this ratio as the optimal condition for preparing active targeting supramolecular nanoscavengers for in vivo experiments. To successfully deliver therapeutic drugs to the lesion site, nanoparticles must first be taken up by the target cells. Therefore, the mechanism of internalization is essential for nanoparticles and their encapsulated drugs, as it is closely linked to their intracellular destiny. Experimental results demonstrated that “cargo exchange” supramolecular nanoscavengers primarily internalize through an energy‐dependently lipid raft‐mediated endocytosis pathway (**Figure** **2**a; Figure S22, Supporting Information). Lipid rafts are dynamic regions within the plasma membrane that are enriched in cholesterol and sphingolipids. The analysis suggested that the internalization of nanoparticles through this pathway was facilitated by the extraction of cholesterol from the plasma membrane by β‐CD present in the nanoparticles.^[^ ^46^ ^]^ Furthermore, this mode of internalization significantly mitigates the degradation of contents by lysosomes, a conclusion corroborated by findings from studies on intracellular fate (Figure S23, Supporting Information). Based on the above discussion, these experimental findings suggested that the modification of the targeting functional element ApoE enhanced the active uptake by target cells, and the lipid raft‐mediated endocytosis pathway reduces the risk of lysosomal degradation, with most reaching the endoplasmic reticulum to release the loaded drugs and exert therapeutic effects.

### 2.4 In Vitro Assessment of Supramolecular Nanoscavengers's Ability to Cross the BBB and Target Tumor Sites

The BBB presents a significant obstacle to the delivery of therapeutic agents to the site of GBM. Therefore, it is crucial to evaluate the ability of supramolecular nanoscavengers to traverse the BBB at the in vitro cellular level. Experimental results demonstrated that modification of the supramolecular nanoscavengers surface with ApoE targeting functional elements significantly enhanced the penetration of nanoparticles through the in vitro BBB model without compromising BBB integrity (Figure 2b,c). This strategy overcome the limitation of the β‐CD macromolecule's inability to cross the BBB, thereby offering the potential for β‐CD to serve as a carrier for therapeutic agents and accumulate at the tumor site. Furthermore, it is clarified that the successfully engineered supramolecular nanoscavenger possessed the capability to traverse the BBB, which was essential for the loaded drugs to attain therapeutic efficacy.

Modification with ApoE targeting functional elements is key to achieving active targeting with supramolecular nanoscavengers. Therefore, we further investigated the targeting ability of supramolecular nanoscavengers at the animal model level. Fluorescence intensity was detected in vivo at different time points after systemic administration for 24 h. The experimental results showed that, compared with supramolecular nanoscavengers without targeting functional element modification, ApoE modification significantly enhanced the accumulation of supramolecular nanoscavengers at the tumor site, with similar trends observed at the ex vivo brain tissue and slice levels (Figure 2d,f; Figure S24, Supporting Information). Furthermore, to more intuitively assess the targeting ability of supramolecular nanoscavengers, the distribution of nanoscavengers was observed in vivo using two‐photon microscopy. The results, as shown in Figure 2g,h, indicated that ApoE‐modified supramolecular nanoscavengers could clearly cross the BBB and accumulated in tumor tissue, whereas supramolecular nanoscavengers without ApoE modification were primarily distributed in blood vessels. These findings strongly suggested that modification with ApoE, as an active targeting functional element, facilitated the ability of supramolecular nanoscavengers to cross the BBB and reach tumor tissue, provided a favorable basis for the treatment of GBM.

### 2.5 In Vitro Cytotoxicity and Mechanisms Investigation of Supramolecular Nanoscavengers

**Figure 3:**
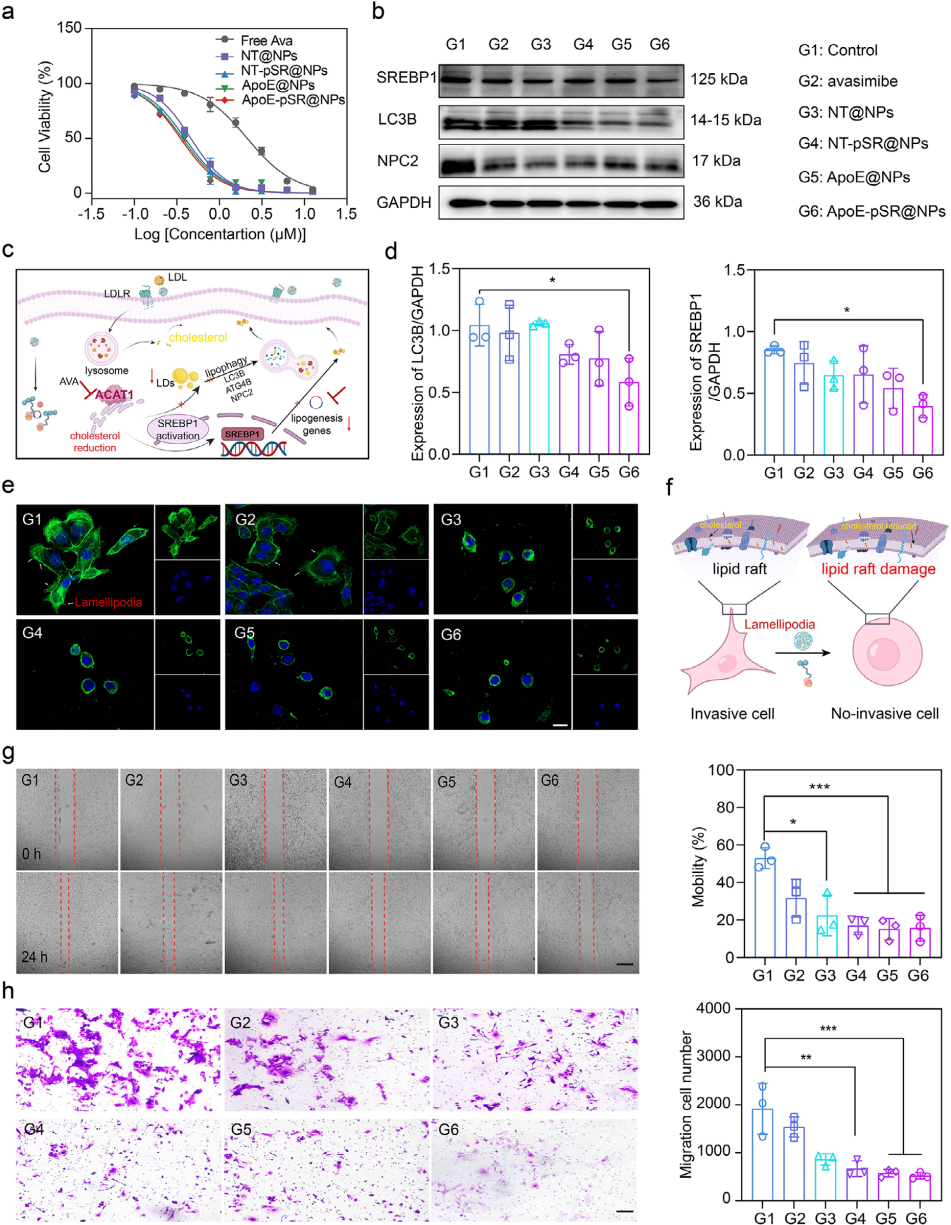
Cellular‐level pharmacodynamic results of supramolecular nanoscavengers. a) IC_50_ fitting curve of supramolecular nanoscavengers (*n* = 3). b–d) Schematic diagram of cholesterol metabolism and key protein WB bands and semi‐quantitative results of supramolecular nanoscavengers (*n* = 3). e,f) Inhibition of pseudopodia formation and schematic diagram of supramolecular nanoscavengers, FITC‐labeled phalloidin staining of pseudopodia, blue represents the cell nucleus, scale bars, 20 µm. g) Migration experiment results of supramolecular nanoscavengers (*n* = 3), scale bars, 100 nm. h) Invasion experiment results of supramolecular nanoscavengers (*n* = 3), scale bars, 100 nm. The data in (d, g, and h) were shown in analytic plots as mean ± s.d., and ordinary one‐way ANOVA was used (*p < 0.05, **p < 0.01, ***p < 0.001, and ****p < 0.0001*)*.

First, the proliferation inhibition of tumor cells by supramolecular nanoscavengers was investigated at the cellular level using the CCK‐8 assay. The experimental results demonstrated that, compared to free AVA, the supramolecular nanoscavengers significantly inhibited the proliferation of tumor cells (**Figure** **3**a), which suggested that after the co‐delivery of the ACAT1 inhibitor AVA and the shSREBP1 gene drug was internalized by tumor cells, the difference in affinity‐where cholesterol has a higher affinity for β‐CD than AVA, which initially occupied the β‐CD cavity for β‐CD, promoted “cargo‐exchange” thereby achieving the release of the loaded drug AVA. Simultaneously, β‐CD functions as both a drug carrier and a cholesterol scavenger. By inhibiting cholesterol storage and its own synthesis, the proliferation of tumor cells was inhibited, which also fully indicated that regulating cholesterol metabolism at the cellular level has a significant killing effect on tumor cells. When the ACAT1 inhibitor AVA reduced free cholesterol esterification to cholesterol, it activated the sterol regulatory element‐binding protein SREBP1, thereby playing a role in its own cholesterol synthesis for cholesterol supplementation. Subsequently, the released shSREBP1 exerted its effects by further diminishing the expression of this gene and inhibiting the supply of endogenous cholesterol. It is undeniable that tumor cells exhibit remarkable adaptability to their environment, when both exogenous and endogenous sources of cholesterol are insufficient, tumor cells will activate the protective lipid autophagy pathway to further supplement cholesterol for maintaining proliferation.^[^ ^36^ ^]^ Therefore, the activation of cholesterol synthesis and the protective lipid autophagy pathway were further investigated. The experimental results showed that, compared to free AVA, the supramolecular nanoscavengers significantly reduced the expression of the key cholesterol synthesis protein SREBP1, which was caused by the effect of shSREBP1. At the same time, the expression of the protective lipid autophagy‐related protein LC3B and NPC2 was also significantly reduced (Figure 3b–d). Based on the above experimental results, these fully indicated that the supramolecular nanoscavengers constructed in this study could inhibit the proliferation of tumor cells by regulating the utilization of cholesterol from multiple perspectives.

Invasive growth is a defining characteristic of GBM, where lamellipodia play a crucial role in the invasion of normal tissues by tumor cells. Lamellipodia have broad, flat protrusions located at the cell periphery that possess the capability to sense the surrounding microenvironment and facilitate cell motility. Specifically, the migration and invasion of tumor cells, along with the formation of lamellipodia, are intricately linked to the integrity of cholesterol‐enriched domains known as lipid rafts.^[^ ^16^, ^46^ ^]^ GBM cells, which have a high proliferation rate, require a continuous supply of cholesterol. The primary function of the nanoscavengers was to regulate cholesterol metabolism from multiple angles. Therefore, we subsequently investigated its impact on lamellipodia. Experimental results confirmed that supramolecular nanoscavengers inhibited cholesterol supply by delivering drugs. Consequently, the extraction of cholesterol from the plasma membrane by β‐CD reduced the concentration of cholesterol in the plasma membrane, thereby inhibiting the formation of lamellipodia (Figure 3e,f), which was conducive to reducing the invasive ability of tumor cells. Subsequently, we assessed the effects of the supramolecular nanoscavengers on tumor cell migration and invasion using scratch and transwell migration assays. The experimental results demonstrated that, compared with the blank control group, the supramolecular nanoscavengers significantly inhibited the migration and invasion of tumor cells (Figure 3g,h), which was mainly attributed to the regulation of cholesterol abundance in cells, thereby affecting biological processes such as membrane biogenesis and invasion. The above experimental results favorably demonstrated that the successfully constructed supramolecular nanoscavengers achieved the release of loaded drugs through the “guest cargo‐exchange” strategy, based on the difference in affinity between the guest molecule and the host molecule β‐CD, and regulated cholesterol metabolism from multiple angles, thereby inhibiting the proliferation and invasion of tumor cells at the cellular level.

### 2.6 Evaluation of Animal‐Level Drug Efficacy and Mechanisms of Supramolecular Nanoscavengers

**Figure 4:**
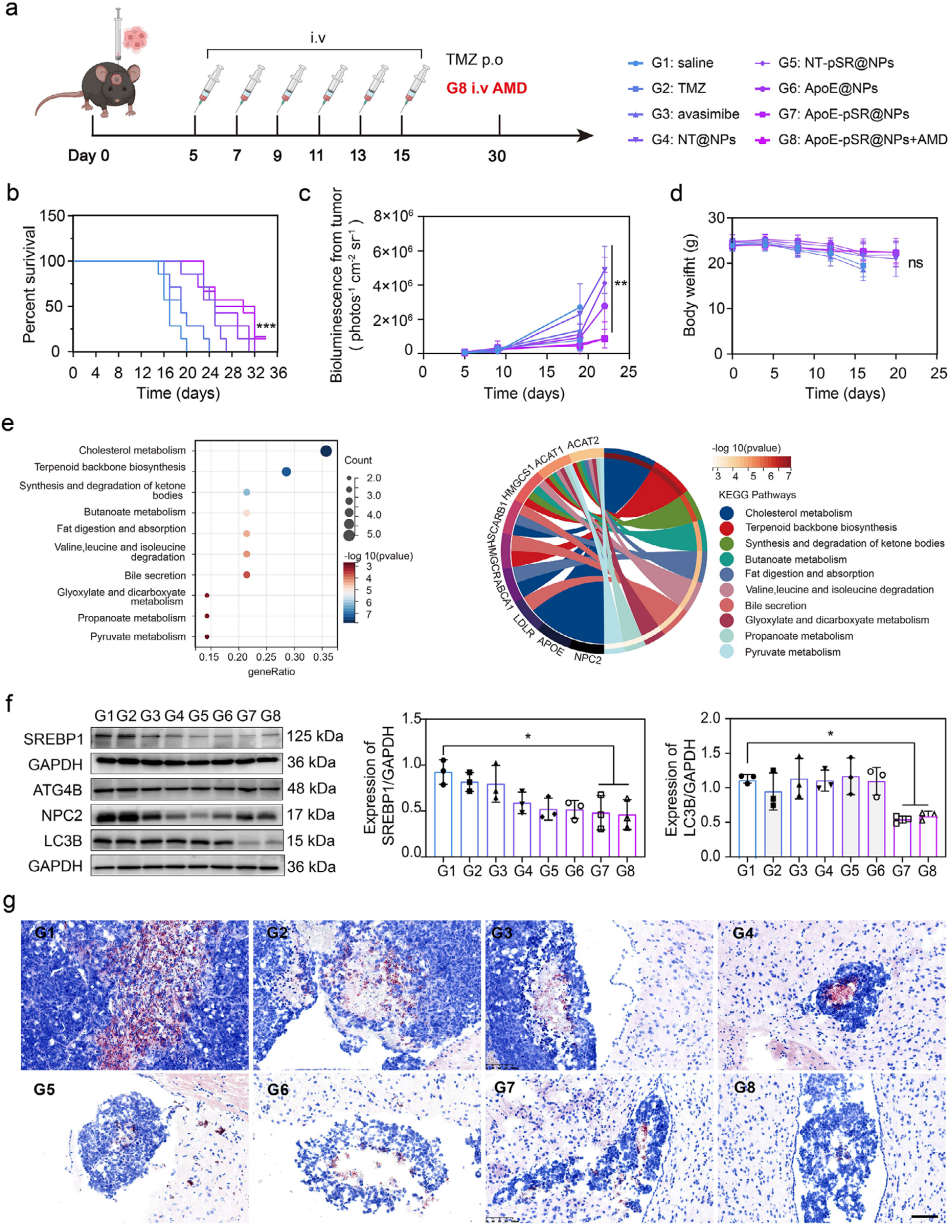
Experimental results of animal efficacy and mechanisms of supramolecular nanoscavengers. a) Establishment and treatment schematic of the GBM orthotopic mouse model. b–d) Survival curves (*n* = 7), tumor luciferase signals (*n* = 4), and body weight of each group (*n* = 4). e) Bubble chart and bioinformatics circle chart of GBM cholesterol metabolism analysis. f) WB bands and semi‐quantitative results of key lipid autophagy proteins of each group (*n* = 3). g) Oil Red O staining results of esterified cholesterol of each group, red represented the Lipid droplets, blue represented nucleus. scale bars, 200 µm. The data in (b, c, and f) are shown in analytic plots as mean ± s.d., and ordinary one‐way ANOVA was used (*p < 0.05, **p < 0.01, ***p < 0.001, and ****p < 0.0001).

To evaluate the therapeutic effect of the “guest cargo‐exchange” supramolecular nanoscavengers, systemic administration was performed in an orthotopic intracranial GBM mouse model. The treatment involved administering the agent every other day for a total of five treatments, with the complete treatment process and drug grouping detailed in **Figure** **4**a. Furthermore, to mitigate potential adverse reactions caused by the released free β‐CD, we administered adamantane intravenously after the completion of the complete clearance agent treatment. Adamantane has a natural affinity for binding with β‐CD, and their interaction helps reduce adverse reactions, particularly the risk of nephrotoxicity.^[^ ^47^ ^]^ The experimental results demonstrated that, compared to the model group, oral temozolomide (Tmz), and the free AVA group, the ApoE peptide‐modified complete formulation significantly prolonged the survival of tumor‐bearing mice (Figure 4b), delayed the increase in tumor signals (Figure 4c; Figure S25, Supporting Information). Moreover, the body weight of tumor‐bearing mice did not significantly decrease during the entire treatment process compared to the untreated group (Figure 4d), further indicated that the supramolecular nanoscavengers exerted its effects without significant toxic side effects. Simultaneously, we conducted a further analysis in conjunction with the TGGA database and found that cholesterol metabolism regulates various pathways closely related to tumor proliferation (Figure 4e). Therefore, based on this, we continued to investigate the expression of key regulatory proteins of cholesterol self‐synthesis, SREBP1, and protective lipid autophagy pathway‐related proteins through WB experiments. The experimental results showed that the gene therapy group and the ApoE peptide‐modified group significantly reduced the expression of SREBP1 and the key lipid autophagy marker protein LC3B (Figure 4f). This was consistent with the trend of the experimental results at the cellular level. Combining the in vivo and in vitro experimental results, it fully demonstrated that the “guest cargo‐exchange” β‐CD‐based supramolecular nanoscavengers successfully delivered the gene drug across the BBB to the tumor site to exert its effect. In addition, we also investigated the distribution of lipid droplets in tumor tissues. Oil Red O staining results showed that, compared to the model group, the gene drug‐loaded and ApoE peptide‐modified formulation groups significantly reduced the distribution of lipid droplets in the tumor site (Figure 4g). Taking together all the above results, it indicated that in the “cholesterol‐rich” microenvironment, the in situ triggered “guest cargo‐exchange” achieved the release of the hydrophobic drug AVA, exerting an inhibitory effect on ACAT1 esterification, thereby reducing lipid droplet distribution.

**Figure 5:**
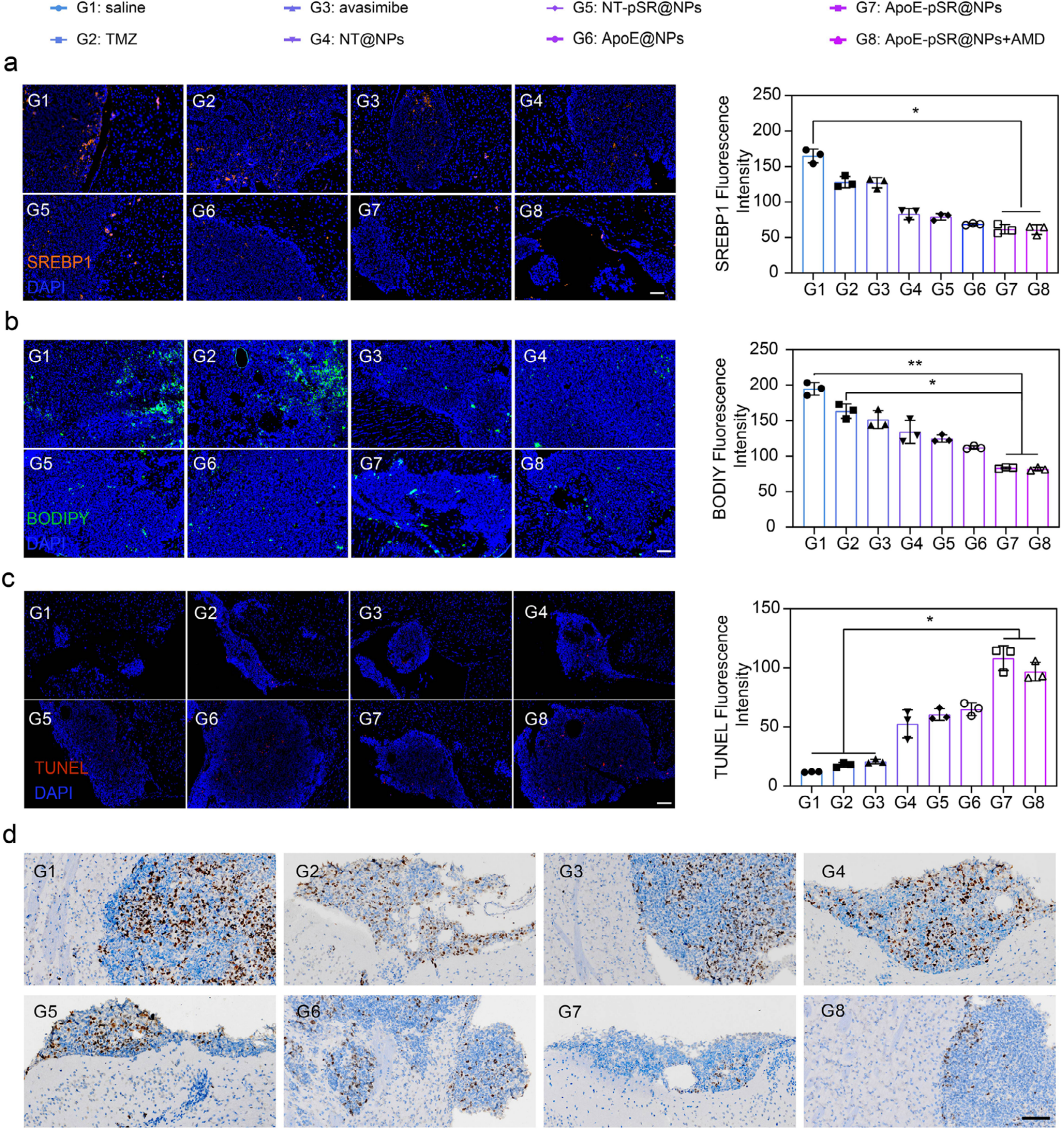
Experimental results of animal pharmacodynamics and mechanisms supramolecular nanoscavengers. a) Immunofluorescence of SREBP1 protein in tumor tissue of each group, orange represented the SREBP1 protein, blue represented the cell nucleus (*n* = 3), scale bars, 200 µm. b) Immunofluorescence of BODIPY (free cholesterol) in tumor tissue of each group, green represented of the BODIPY (free cholesterol), blue represented the cell nucleus (*n* = 3), scale bars, 200 µm. c) TUNEL results image of each group, red represented the TUNEL, blue represented the cell nucleus (*n* = 3), scale bars, 200 µm. d) ki 67 results image in tumor tissue of each group, Brown represented the ki67, blue represented the cell nucleus, scale bars, 200 µm. The data in (a–c) were shown in analytic plots as mean ± s.d., and ordinary one‐way ANOVA was used (*p < 0.05, **p < 0.01, ***p < 0.001, and ****p < 0.0001).

Following the completion of the comprehensive treatment, the expression of the cholesterol self‐synthesis regulatory protein SREBP1 was examined at the tissue section level using immunofluorescence. The experimental results demonstrated that, compared to the model group, the oral TMZ group, and the free AVA group, both the gene‐loaded drug and the complete formulation group significantly reduced the expression of the SREBP1 protein (**Figure** **5**a), which was consistent with the results of the aforementioned WB experiment. Furthermore, the distribution of free cholesterol in tumor tissues was investigated using immunofluorescence, primarily by labeling free cholesterol with the BODIPY probe. The experimental results indicated that, compared to the model group and the free drug group, the complete formulation group significantly reduced the distribution of free cholesterol in tumor tissues (Figure 5b). These experimental findings suggested that the in situ‐triggered “cargo‐exchange” supramolecular nanoscavengers, through the displacement of excess free cholesterol in the microenvironment, releases the therapeutic drug AVA and shSREBP1. This process inhibited cholesterol esterification, reduced the activation of self‐cholesterol synthesis due to insufficient exogenous cholesterol uptake by tumor cells, and simultaneously inhibits the activation of the protective lipid autophagy pathway, thereby achieving the goal of inhibiting tumor proliferation through multi‐faceted regulation of cholesterol metabolism. In addition, the tumor proliferation index and apoptosis of mice after drug treatment were further examined using both immunohistochemistry and immunofluorescence methods. The experimental results are shown in Figure 5c,d, with the gene‐loaded drug group and the complete formulation group significantly increasing tumor cell apoptosis and reducing abnormal tumor cell proliferation. In summary, the evaluation of treatment efficacy at the animal level has clearly demonstrated that the “cargo‐exchange” supramolecular nanoscavengers could effectively inhibit GBM proliferation through the regulation of cholesterol metabolism.

### 2.7 Investigation of the Remodeling of the Immune Microenvironment by “Cargo‐Exchange” Supramolecular Nanoscavengers

**Figure 6:**
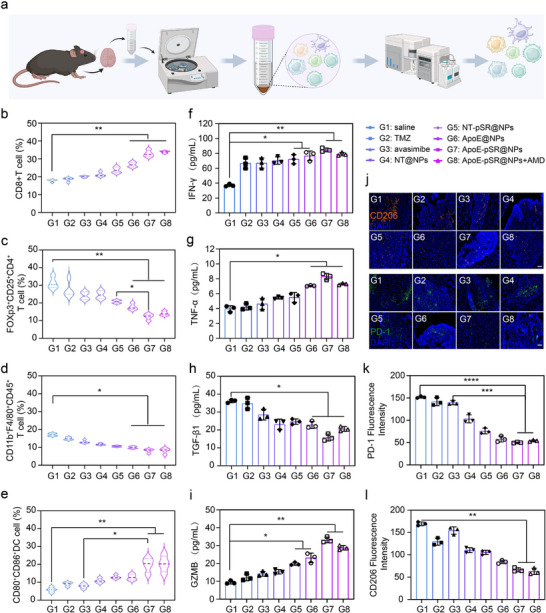
Experimental results of animal pharmacodynamics and mechanisms of supramolecular nanoscavengers. a) Schematic diagram of Flow Cytometric analysis of immune cells in tumor tissue. b) Tumor tissue CTL analysis of each group (*n* = 4). c) Tumor tissue Treg cells analysis of each group (*n* = 4). d) Tumor tissue microglia/macrophages analysis of each group (*n* = 4). e) Lymph node DC cells analysis of each group (*n* = 4). f–i) results of immune factors (IFN‐γ, TNF‐α, TGF‐β1, and GZMB) (*n* = 3). j–l) Immunofluorescence and semi‐quantitative results of macrophages (CD206) and exhausted T cells (PD‐1) of each group, orange represented CD206, green represented PD‐1, and blue represented the nucleus (*n* = 3), scale bars, 200 µm. The data in (b–i and k,l) are shown in analytic plots as mean ± s.d., and ordinary one‐way ANOVA was used (*p < 0.05, **p < 0.01, ***p < 0.001, and ****p < 0.0001*)*.

According to the literature, dysregulation of cholesterol metabolism in GBM further exacerbates the development of an immunosuppressive microenvironment, thereby facilitating the invasion and proliferation of tumor cells.^[^ ^21^ ^]^ Mice with orthotopic intracranial GBM models were sacrificed after three intravenous injections of the drug for treatment. Tumor tissues and cervical lymph nodes were prepared into single‐cell suspensions, and the distribution of various immune cells was investigated by flow cytometry (**Figure** **6**a). The experimental results showed that, compared with the model group, the gene‐loaded drug group and the complete formulation group could significantly increase the distribution of cytotoxic T lymphocytes in the tumor site (Figure 6b; Figure S26, Supporting Information), which is a key indicator for the immune system to exert a direct killing effect on GBM cells.^[^ ^48^ ^]^ At the same time, the distribution of regulatory T cells with significant immunosuppressive function in tumor tissues was reduced (Figure 6c; Figure S27, Supporting Information), and the infiltration of immunosuppressive M2‐type microglia/macrophages in the tumor site was reduced (Figure 6d; Figure S28, Supporting Information). Similarly, the activation ratio of DC cells in the cervical lymph nodes was increased (Figure 6e; Figure S29, Supporting Information). DC cells are key indicators for presenting antigens to T cells and activating their killing effect, indicating that peripheral immunity was activated and infiltrated into the central tumor site after treatment. In addition, we also investigated the activation of cytotoxic T cells in the spleen. The experimental results showed that the complete formulation group also significantly increased the proportion of CTL cells in the spleen (Figures S30 and S31, Supporting Information). This further indicated that after treatment, the inhibitory immune microenvironment in the tumor site was corrected, and at the same time, peripheral immunity was activated, and the infiltration into the tumor site was increased. In addition, the expression of immune‐related cytokines was investigated by ELISA kits. The experimental results showed that after treatment, compared with the model group, the complete formulation group significantly increased the secretion of IFN‐γ, TNF‐α, and granzyme B, and reduced the secretion of the inhibitory cytokine TGF‐β (Figure 6f–i). Finally, at the tissue slice level, the distribution of inhibitory macrophages and Treg cells was further investigated by immunofluorescence. The experimental results were consistent with the flow cytometry results. The complete formulation group could significantly reduce the infiltration of M2‐type macrophages in the tumor site, and at the same time, the proportion of exhausted T cells were also reduced (Figure 6j–l), the above results indicated that the “cholesterol‐rich” pressure in the microenvironment helps to reduce the over‐expression of exhausted phenotypes of cytotoxic T cells, thereby weakening the killing effect on tumor cells. In summary, the multi‐dimensional regulation of dysregulated cholesterol metabolism in GBM by supramolecular nanoscavengers, along with the removal of excess cholesterol from the microenvironment, facilitated the remodeling of a suppressive immune microenvironment, thereby enhancing the cytotoxic effects on tumor cells.

### 2.8 Safety Evaluation of “Cargo‐Exchange” Supramolecular Nanoscavengers

Developing an in situ “cargo‐exchange” supramolecular nanoscavengers for tumors with good biosafety is the key to exerting the therapeutic effect. After five treatments, the mice in each treatment group were tested for blood routine biochemical indicators and the safety of major organs to evaluate the biosafety of the “cargo‐exchange” nanoscavengers. Specifically, following the completion of the treatment, to mitigate the toxic and side effects associated with the cholesterol extraction of free β‐CD on normal cells, the G8 group received an additional injection of amantadine (AMD), which is capable of forming a stable supramolecular complex with β‐CD and acts as an antidote agent against these toxicities and side effects. The experimental results showed that each treatment group had good biosafety (Figures S32 and S33, Supporting Information). These also fully indicated that the modification of β‐CD with hydrophilic poly‐amino‐acid chains and the pre‐occupation of the cavity by hydrophobic drugs helped to improve the water solubility of β‐CD, realized the possibility of delivering drugs through the BBB of central nervous system, and reduced the hemolysis risk of β‐CD. In addition, we further investigated the safety of the supramolecular nanoscavenger on normal brain tissue in healthy mice under the same treatment cycle and dosage. The experimental results indicated that during this treatment phase, the core component β‐CD of the supramolecular nanoscavengers did not elicit any significant toxic or adverse effects on normal brain tissue (Figure S34, Supporting Information). In conclusion, the supramolecular nanoscavengers developed in this study based on cargo‐exchange mechanisms demonstrated significant therapeutic efficacy and biological safety, which provided favorable evidence for β‐CD as a drug carrier and cholesterol scavenger through vascular administration.

## 3 Conclusion

In summary, we have successfully developed the “cargo‐exchange” supramolecular nanoscavengers that could be activated within the “cholesterol‐rich” microenvironment of GBM. This achievement was facilitated by leveraging the natural affinity between the key endogenous lipid molecule, cholesterol, and β‐CD, which surpassed the affinity between the hydrophobic therapeutic agent AVA and β‐CD. By hydrophilically modifying β‐CD and developing a nanoclearing agent, it was possible to regulate the cholesterol metabolism of tumor cells from multiple perspectives, thereby inhibiting tumor proliferation. Concurrently, the released β‐CD performed a dual function as both a carrier and a cholesterol scavenger, effectively removing excess cholesterol from the tumor microenvironment. This action alleviated the burden on immune cells and rectified the immunosuppressive microenvironment, thereby activating immune function and enhancing the anti‐tumor response. In conclusion, this study provided a promising application for β‐CD in GBM and conditions associated with central cholesterol overload. Additionally, there remain avenues for further investigation, particularly regarding the specific mechanisms by which based “cargo‐exchange” supramolecular nanoscavengers remodels the immunosuppressive microenvironment.

## 4 Experimental Section

### Chemicals and Materials

All chemicals utilized in the synthesis were of analytical grade and were employed in accordance with the manufacturer's recommendations. N'‐tert‐Butyloxycarbonyl‐L‐ornithine (purity >98%), N6‐Cbz‐L‐Lysine (purity >98%), tri‐phosgene (purity >98%), trifluoroacetic acid, and hydrobromic acid were purchased from Adams (Shanghai, China). mPEG‐NH_2_ (purity >98%) was purchased from Xiao Biological Technology Co., Ltd. (Xi'an, China). β‐Cyclodextrin (purity >98%) was purchased from Merck. 3,5‐Dimethylpyrazole‐1‐carboxamidine nitrate (purity >98%) and sodium chloroacetate (purity >98%) were purchased from Aladdin (Shanghai, China). BODIPY493/503 was purchased from MCE (USA, CAS No:121207‐31‐6). Avasimibe (purity >99%) was purchased from Selleck (Shanghai, China, CAS: No 166518‐60‐1). shSREBP1 was purchased from Gene Pharma (Shanghai, China). CCK‐8 (Cell Counting Kit‐8) and D‐luciferin potassium salt were purchased from Melian Biotechnology (Shanghai, China). Phalloidin‐FITC was purchased from Abcam (ab235137). Flow cytometry antibodies were purchased from Abcam. Methanol and acetonitrile (chromatographic grade), as well as dialysis bags (MWCO = 3.5 kDa/5 kDa/7 kDa/10 kDa), were purchased from Thermo Fisher Scientific (USA). SREBP‐1 antibody was purchased from SANTA CRUZ biotechnology (sc‐365513), LC3B (A11282), ATG4B (A5059), NPC2 (A5413), and ACAT1 (A13273) antibodies were purchased from ABclonal.

### Cells and Animals

GL261 GBM cells, GL261‐luc+ cells, and GL261‐luc+green+ cells were purchased from Shanghai Gene Chem Co., Ltd. (Shanghai, China). Male C57BL/6 mice (6–8 weeks old) were purchased from Shanghai Bi Kai Experimental Animal Co., Ltd. (Shanghai, China). All animal experiments conducted in this study received approval from the Experimental Animal Ethics Committee of the School of Pharmacy at Fudan University and adhered strictly to the relevant management regulations (2022‐07‐YJ‐JC‐86).

The patient‐derived samples utilized in this study were obtained from the General Hospital in Shanghai. The collection of these samples was approved by the Ethics Committee of General Hospital (Approval No.:2019S211), and informed consent was secured from the participating patients.

### Establishment and Validation of the Murine Orthotopic GBM Model

GL261‐luc+ cells were subjected to digestion, followed by centrifugation and collected, which were subsequently injected at a concentration of 5 µL containing 3 × 10^5 cells per mouse, positioned 2 mm to the right of the bregma and at a depth of 3 mm. On day 15, the mice were euthanized and harvested brain tissues. The expression levels of the cholesterol esterification enzyme ACAT1 and the sterol regulatory element‐binding protein SREBP1 were assessed using Western blotting (WB) and immunofluorescence techniques. Additionally, free cholesterol and lipid droplets were analyzed through immunofluorescence and Oil Red O staining.

### Acquisition and Validation of the Patient‐Derived Samples

Tumor and adjacent normal tissue samples from patients with glioblastoma (GBM) were procured from General Hospital in Shanghai. The expression levels of the cholesterol esterification enzyme acyl‐CoA: cholesterol acyltransferase 1 (ACAT1) and the sterol regulatory element‐binding protein 1 (SREBP1) were assessed using WB and immunofluorescence techniques. Additionally, free cholesterol and lipid droplets were analyzed through immunofluorescence and Oil Red O staining methods.

### Synthesis and Characterization of β‐CD‐Based Polymers

Synthesis of compound 1: 1 g of N'‐tert‐Butyloxycarbonyl‐L‐ornithine (4.31 mmol, 1 equiv.) and 0.5 g of triphosgene (1.72 mmol, 0.4 equiv.) of triphosgene were accurately weighed and subsequently added to 50 mL of anhydrous tetrahydrofuran (THF). The reaction flask was purged with argon gas, and the mixture was stirred in an oil bath maintained at 50 °C. The reaction was deemed complete when the solution transitioned from a suspension to a clear liquid. Following this, the reaction mixture was allowed to cool to room temperature and was then filtered to eliminate any unreacted solid materials. The resulting filtrate was gradually introduced to pre‐cooled n‐hexane while stirring, facilitating the precipitation of the desired product. The mixture was then stored at −20 °C overnight, after which the precipitate was collected via vacuum filtration using a Buchner funnel. The filter cake was subsequently dried under vacuum, and a small portion of the resulting white solid product was reserved for ^1^HNMR analysis.

### Synthesis of Compound 2

1 g of N6‐Cbz‐L‐Lysine (3.57 mmol, 1 equiv.) and 0.42 g (0.57 mmol, 0.4 equiv.) triphosgene were accurately weighed and subsequently added to 50 mL of anhydrous tetrahydrofuran (THF). The reaction flask was purged with argon gas, and the mixture was stirred in an oil bath maintained at 50 °C for a duration of 3 h. During this period, the reaction mixture transitioned from a suspension to a clear solution. Following the completion of the reaction, the solution was allowed to cool to room temperature and was then filtered to eliminate any unreacted solid materials. The resulting filtrate was gradually added to pre‐cooled n‐hexane while stirring, facilitating the precipitation of the desired product. The mixture was subsequently stored at −20 °C overnight, after which the precipitate was collected via vacuum filtration using a Buchner funnel. The filter cake was dried under vacuum, and a small portion of the resulting white solid product was reserved for ^1^HNMR analysis.

### Synthesis of Compound 3

30 mg (1 equiv.) of PEG500‐NH2, 155 mg of Compound 1 (8 equiv.), and 165 mg of Compound 2 (8 equiv.) were accurately measured and subsequently added to 20 mL of anhydrous dimethylformamide (DMF). The reaction flask was purged with argon gas, and the resulting mixture was stirred in an oil bath maintained at 50 °C for a duration of 48 h. Following the completion of the reaction, the product underwent dialysis (molecular weight cutoff = 3.5 kDa) and was subsequently lyophilized to yield a white powder. A small aliquot of the product was reserved for ^1^HNMR analysis.

### Synthesis of Compound 4

200 mg of Compound 3 was introduced into anhydrous dichloromethane/trifluoroacetic acid (DCM/TFA) in a 1:1 volume ratio and allowed to react on ice for a duration of 4 h. The solvent mixture was subsequently removed via rotary evaporation under reduced pressure. Following this, 375 mg of 3,5‐dimethylpyrazole‐1‐carboximidamide nitrate was added, along with 15 mL of deionized water. The pH of the solution was adjusted to 9.3 using 1 M sodium hydroxide (NaOH), and the reaction was conducted at 37 °C for 48 h. Upon completion of the reaction, the product underwent dialysis (molecular weight cutoff = 3.5 kDa) and was lyophilized to yield a white powder. A small aliquot of the product was reserved for ^1^HNMR analysis.

### Synthesis of Compound 5

Compound 4 was dissolved in 5 mL of TFA, and 0.5 mL of HBr/HOAc solution (33%, v/v) was added while maintaining the reaction mixture in an ice bath. The reaction flask was sealed, and the mixture was allowed to react for a duration of 3 h. Following this, a portion of the solvent was removed via rotary evaporation under reduced pressure. The resulting reaction solution was subsequently enclosed in a dialysis bag with a molecular weight cutoff (MWCO) of 3.5 kDa and dialyzed in deionized water for 48 h. The final product was lyophilized to yield a white solid, from which a small sample was taken for ^1^HNMR analysis.

### Synthesis of Compound 6

2.5 g of β‐CD was introduced into 100 mL of NaOH solution at a temperature of 0 °C while being continuously stirred. The mixture was subsequently heated to 90 °C and maintained at this temperature for a duration of 1 h. Following this, 3.53 g of sodium chloroacetate were incorporated into the solution, which was stirred for an additional 3 h. The reaction mixture was then allowed to cool to room temperature, and the pH was adjusted to 6 using hydrochloric acid. The resultant product was precipitated using a methanol/ethanol mixture in a 3:1 volume ratio and was subsequently dried under vacuum to yield a white solid. A small aliquot of the product was reserved for ^1^HNMR analysis and Fourier transform infrared spectroscopy (FTIR).

### Synthesis of Compound 7

41 mg (0.21 mmol) of Compound 7 and 79.8 mg (0.21 mmol) of HATU were accurately weighed and subsequently introduced into a two‐necked flask under an atmosphere of high‐purity argon. Following this, 5 mL of anhydrous DMSO was added to facilitate the activation of the carboxyl group at room temperature. After a reaction period of 1 h, 200 mg (0.03 mmol) of Compound 5, which had been previously dissolved in DMSO, along with 15.5 mg (0.12 mmol) of DIPEA, were incorporated into the reaction mixture. The reaction was allowed to proceed at room temperature for 24 h under the same high‐purity argon conditions. Upon completion of the reaction, the resulting solution was transferred to a dialysis bag (MWCO = 3.5 kDa) and dialyzed in deionized water for a duration of 48 h. The final product was then lyophilized to yield a white solid, from which a small aliquot was taken for ^1^HNMR analysis.

### Synthesis of Compound 8

12 mg (1 equiv.) of Compound 7 and 13 mg (1 equiv.) of MAL‐PEG3.5K‐NHS were accurately measured and subsequently dissolved in 10 mL of phosphate‐buffered saline (PBS) at a pH of 7.0. The reaction was conducted at a temperature of 37 °C for 1 or 2 h. Following the reaction, the resultant product was subjected to dialysis in deionized water (MWCO = 5 kDa) for 48 h, after which it was lyophilized to yield a white solid. A small aliquot of the final product was reserved for ^1^HNMR analysis.

### Synthesis of Compounds 9 and 10

30 mg (1 equiv.) of Compound 8 and 12.82 mg (1 equiv.) of MAL‐PEG3.5K‐NHS were accurately measured and subsequently dissolved in 10 mL of PBS (pH 7.0). The reaction was conducted at a temperature of 37 °C for a duration of 1 h to yield Compound 9. Following this, 10 mg (1.2 equiv.) of the ApoE peptide was introduced directly into the reaction mixture, and the reaction was allowed to proceed for an additional 24 h. The resultant product was subjected to dialysis in deionized water (MWCO = 7 kDa) for 48 h, after which it was lyophilized to obtain a white solid. A small aliquot of the final product was reserved for ^1^HNMR analysis.

### Determination of Critical Aggregation Concentration of β‐CD‐Based Polymer

The critical aggregation concentration (CAC) of polymers or β‐CD with varying degrees of polymerization was determined utilizing pyrene as a fluorescence indicator. The polymers and β‐CD were diluted to different concentrations. Subsequently, 999 µL of each polymer solution was combined with 1 µL of a pyrene acetone solution (0.1 mm) and incubated at 37 °C overnight. The fluorescence spectra of pyrene in the various nanoparticle dilutions were recorded using a microplate reader (Excitation, Ex═335 nm), and the fluorescence intensity was measured at an emission wavelength of 384 nm and subsequently plotted.

### Preparation of Supramolecular Nanoscavengers by Nanoprecipitation

AVA and the polymer were dissolved in dimethyl sulfoxide (DMSO) at a mass ratio of 1:10, which served as the organic phase. The mixture was stirred at room temperature overnight. Subsequently, the solution was added dropwise to either pure water or PBS to facilitate the formation of nanoscavengerss under the influence of a magnetic field. The DMSO was subsequently removed through dialysis, and the particle size, drug loading, and encapsulation efficiency were subsequently assessed.

### Characterization of Supramolecular Nanoscavengers

The optimal binding ratio of shSREBP1 to the nanoscavengers was determined using agarose gel electrophoresis. The morphology of the nanoscavengers was examined by transmission electron microscopy (TEM), and the surface element analysis was conducted via Energy‐dispersive X‐ray spectroscopy (EDS). The release behavior of AVA and shSREBP1 plasimd from the nanoscavengers were assessed using the dialysis bag method. Meanwhile, the stability of the supramolecular nanoscavengers was assessed under two distinct conditions: storage at 4 °C and a simulated physiological environment comprising 10% FBS at 37 °C. Additionally, the biocompatibility of the nanoscavengers was evaluated through in vitro hemolysis experiments.

### 4.1 Nuclear Magnetic Resonance ROESY Characterization of Supramolecular Nanoscavengers

Nanoscavengers agents encapsulating cholesterol and AVA were prepared separately by nanoprecipitation. After freeze‐drying, the hydrogen‐hydrogen interactions of different cargo molecules were characterized by Rotating Frame Overhauser Effect Spectroscopy (ROESY).

### Molecular Docking

The docking software AutoDock Vina was utilized, with the docking method configured to ″set Rigid filename A total of 50 docking runs were performed to analyze the binding interactions between cholesterol‐β‐CD and AVA‐β‐CD and calculate the binding energy.

### Screening of ApoE Targeting Functional Element Modification Ratio for Supramolecular Nanoscavengers

The determination of the ApoE modification ratio for the targeting of functional elements was conducted through the preparation of supramolecular nanoscavengers with varying ApoE modification ratios encapsulating DID, utilizing the nano‐precipitation method, where the size and zeta potential were measured by DLS. These nanoscavengers were subsequently co‐cultured with GL261 cells for a duration of 1 h. The fluorescence intensity of DID within the tumor cells was examined by flow cytometry to determine the optimal modification ratio of the ApoE targeting functional elements.

### Investigation of Cellular Uptake Pathway and Intracellular Fate of Supramolecular Nanoscavengers

GL‐261 cells were cultured in 12‐well plates at a density of 10^^5^ cells per well. The growth status was monitored via microscopy until cell confluence reached ≈70–80%. Subsequently, the cells were co‐incubated with DID‐labeled nanoscavengerss for 60 min, followed by three washes with Hank's Balanced Salt Solution (HBSS). The cells were then stained with Lyso/ER/Golgi tracker at 37 °C for 45 min, washed three times with HBSS, and subsequently stained with Hoechst at 37 °C for 15 min. Observations were conducted using a confocal microscope.

### Investigation of the Ability of Supramolecular Nanoscavengers to Cross the BBB In Vitro

Bend.3 cells were seeded at a density of 10^5 cells per well in transwell chambers and cultured for a duration of 14 days. To evaluate the integrity of the BBB model, FITC‐labeled dextran was introduced into the inserts. The cumulative diffusion volume (V) at this time point was calculated by dividing the cumulative diffusion amount of FITC‐dextran in the receiving pool at different time points and the initial concentration of FITC‐dextran in the supply pool. A linear regression line was performed with V as the ordinate and time (t) on the abscissa, and its slope of this regression line represents the clearance rate (P), which corresponds to the permeability coefficient of FITC‐dextran in the in vitro BBB model. The capacity of the fluorescently labeled nanoscavengerss to traverse the BBB model was assessed by adding them to the inserts. Samples from the receiving cells were collected at 60 min, and the fluorescence intensity was measured to determine the efficiency of the nanoscavengers in crossing the BBB.

### Investigation of the Targeting of Supramolecular Nanoscavengers to GBM

GL261‐luc+ cells were subjected to digestion, followed by centrifugation and collection. Subsequently, these cells were injected into the right side of the mouse's anterior fontanelle at a depth of 3 mm and a distance of 2 mm, with a dosage of 5 µL containing 300 000 cells per mouse. On day 12, formulations of DID‐labeled NT‐pSR@NPs and ApoE‐pSR@NPs were administered via the tail vein. The targeting efficacy of the nanoscavengers to GBM was assessed at various time points utilizing small animal in vivo imaging and two‐photon microscopy.

### In Vivo Targeting of Supramolecular Nanoscavengers to GBM with Two‐Photon Microscopy

GL261‐luc+ cells were subjected to digestion, followed by centrifugation and collection. A volume of 5 µL containing 3 × 10^^5^ cells per mouse was injected into the right side of the mouse's bregma, specifically 2 mm lateral and at a depth of 3 mm. On day 12, formulations of NT‐pSR@NPs and ApoE‐pSR@NPs, labeled with DID, were administered via the tail vein. The targeting efficacy of the nanoscavengers to GBM was assessed using small animal in vivo imaging and two‐photon microscopy at different time points.

### Cells Proliferation Inhibition

GL‐261 cells were cultured in 96‐well plates at a density of 5 × 10^^4^ cells per well. The growth status was monitored via microscopy until cell confluence reached ≈70–80%. Subsequently, the cells were treated with equivalent concentrations of free AVA, NT@NPs, NT‐pSR@NPs, ApoE@NPs, and ApoE‐pSR@NPs, followed by co‐incubation for 48 h. After this period, a 10% CCK‐8 solution was added, and the cells were incubated for an additional hour before measuring the absorbance at 450 nm.

### Observation of Invasive Pseudopodia

GL‐261 cells were cultured in 12‐well plates at a density of 10^^5^ cells per well. The growth status was monitored via microscopy until cell confluence reached ≈70–80%. The cells were subsequently treated with equivalent concentrations of AVA, NT@NPs, NT‐pSR@NPs, ApoE@NPs, and ApoE‐pSR@NPs, alongside a blank control group. Following a 24‐h co‐incubation period, phalloidin‐FITC and DAPI staining were conducted, and observations were made using confocal microscopy.

### Migration Ability

GL261 cells were seeded in six‐well plates at a density of 105 cells per well until the cell confluence reached 100%. The cells were scratched using the tip of a P200 pipette. The detached cells were washed away with HBSS. The cells were treated with the same concentrations of AVA, NT@NPs, NT‐pSR@NPs, ApoE@NPs, and ApoE‐pSR@NPs, and a blank control group was set. After incubation at 37 °C for 48 h, images of the scratch area were captured and collected at 48 h using an inverted phase‐contrast fluorescence microscope.

### Invasion Ability

Pre‐cooled Matrigel, diluted at a ratio of 1:8 with basal medium, was applied to a 6.5 mm polycarbonate membrane insert featuring 8.0 µm pores. This insert was subsequently placed into a 24‐well plate and incubated at 37 °C overnight in an incubator. Prior to the experiment, GL261 cells were subjected to a 12‐h starvation period using serum‐free basal DMEM/F12 medium. The cells were then digested, centrifuged, and the supernatant was discarded. Following this, the cells were resuspended in serum‐free medium. A total of 5 × 10^4 cells were seeded in the upper chamber (200 µL) that had been coated with Matrigel. The medium in the upper chamber consisted of DMEM/F12 basal medium devoid of FBS, along with the corresponding treatments (Free AVA, NT@NPs, NT‐pSR@NPs, ApoE@NPs, ApoE‐pSR@NPs), while a blank control group was also established. The medium in the lower chamber contained DMEM/F12 medium supplemented with 20% FBS (750 µL). After a 48‐h culture period, the medium in the upper chamber was discarded, and the cells were washed three times with HBSS. The cells were then fixed using 4% paraformaldehyde (PFA) at 4 °C for 5 min and subsequently stained with a 0.1% crystal violet solution for 10 min, followed by five washes with distilled water. The cells in the upper chamber were removed using a cotton swab, and the remaining cells were washed three additional times with HBSS. Finally, images of the lower surface of the upper chamber were captured using an inverted phase‐contrast microscope.

### Investigation of Lipid Autophagy Pertinent Proteins

GL261 cells were cultured in six‐well plates at a density of 10^^5^ cells per well. Following cell adhesion, the cells were treated with various formulations, including Free AVA, NT@NPs, NT‐pSR@NPs, ApoE@NPs, and ApoE‐pSR@NPs, alongside a blank control group. After a 24‐h incubation period at 37 °C, the cells were lysed, and the supernatant was collected for analysis. The expression of lipid autophagy proteins, specifically SREBP1, LC3B, and NPC2, was assessed using Western blotting techniques.

### Therapeutic Effects of Supramolecular Nanoscavengers on GBM Models

GL261‐luc+ cells were subjected to digestion, followed by centrifugation and collection. A volume of 5 µL containing 3 × 10^^5^ cells per mouse was injected into the right side of the mouse's bregma, specifically 2 mm lateral and at a depth of 3 mm. On day 5, the mice were randomly assigned to various groups: model group, TMZ (administered orally), free AVA, NT@NPs, NT‐pSR@NPs, ApoE@NPs, ApoE‐pSR@NPs, and ApoE‐pSR@NPs combined with amantadine (AMD). The drugs were administered via the tail vein every 2 days, and parameters such as body weight, tumor luciferase signal, and survival curves were monitored every 4 days.

### Investigation of the Mechanisms of the Immune Nicroenvironment by “Cargo‐Exchange” Supramolecular Nanoscavenger

Following the fifth administration, the mice from each group were euthanized. Brain tissue and serum samples were collected for analysis. The expression of lipid autophagy proteins, the distribution of free cholesterol and lipid droplets, as well as organ safety, were assessed using immunofluorescence, Western blotting, Oil Red O staining, and hematoxylin and eosin (HE) staining techniques.

### Investigation of the Remodeling of the Immune Microenvironment by “Cargo‐Exchange” Supramolecular Nanoscavenger

Following the third administration, a total of four mice per group were euthanized. Brain tissue, spleen, and cervical lymph nodes were harvested and processed into single‐cell suspensions. Immune cells were isolated from the brain tissue utilizing Percoll density gradient centrifugation. The isolated cells were subsequently stained with antibodies specific for flow cytometry antibodies to investigate the infiltration of CD8^+^ T cells, dendritic cells (DCs), regulatory T cells (Tregs), and macrophages. The presence of programmed cell death protein 1 (PD‐1) and M2 macrophages was evaluated through immunofluorescence at the tissue section level. Additionally, cytokine levels were quantified using enzyme‐linked immunosorbent assay (ELISA) kits.

### In Vivo Safety Evaluation of Supramolecular Nanoscavengers

Following treatment, the in vivo safety of each formulation was evaluated in a murine model of orthotopic intracranial GBM. This assessment involved measuring serum biochemical parameters and conducting hematoxylin and eosin (H&E) staining of critical organs, including the heart, liver, spleen, lung, and kidney.

### Safety Assessment of Normal Brain Tissue

To assess the safety of β‐CD in supramolecular nanoscavengers for normal tissues, NT@NPs, NT‐pSR@NPs, ApoE@NPs, and ApoE‐pSR@NPs were administered via intravenous injection into the tail veins of healthy mice every 2 days for a total of five administrations. Subsequently, the impact of β‐CD on normal brain tissue was evaluated through HE staining of brain tissue specimens.

### Statistics

All statistical analysis was conducted employing GraphPad Prism 9 software, and data were presented as mean ± s.d. Utilizing the unpaired *t*‐test for comparing between two groups and ordinary one‐way analysis of variance (ANOVA) test for comparing between more groups. Two‐way ANOVA test was applied for the tumor growth curves. Log‐rank test was applied for difference in the survival curve. Significant differences were indicated by *p < 0.05, **p < 0.01, ***p < 0.001 and ****p< 0.0001.

## Conflict of Interest

The authors declare no conflict of interest.

## Supporting information



Supporting Information

## Data Availability

The data that support the findings of this study are available from the corresponding author upon reasonable request.
