# Supplementary material for: A Supramolecular Nanoscavengers: Based “Cargo‐Exchange” Breaking Cholesterol Metabolic Dysregulation for Glioblastoma Therapy
Source: Adv Sci (Weinh). 2025 Nov 20;13(7):e19690. doi: 10.1002/advs.202519690 (PMC12866777; doi:10.1002/advs.202519690)
Supplement: Supplementary file 1 — Supporting Information [file ADVS-13-e19690-s001.pdf]

Supporting Information for

A Supramolecular Nanoscavengers: based "Cargo-Exchange" Breaking Cholesterol Metabolic Dysregulation for Glioblastoma Therapy

Zonghua Tian[1], *Yun Chen*[1], *Jingyi Zhou*[1], *Shilin Zhang*[1], *Hongrui Fan*[1], *Xuwen Li*[1], *Tao Sun*[1,*], *Chen Jiang*[1,*]

[1]*Department of Pharmaceutics School of Pharmaceutical Sciences, Fudan University, Key Laboratory of Smart Drug Delivery Ministry of Education, State Key Laboratory of Brain Function and Disorders, Shanghai 201203, China*

*: *corresponding author*

E-mail: sunt@fudan.edu.cn; jiangchen@shmu.edu.cn

Contents:

Figure S1. Validation of cholesterol reprogramming characteristics in a murine GBM models

Figure S2. Validation of cholesterol reprogramming characteristics in human GBM sample

Figure S3. Synthetic Route for Brush-like Poly (amino acid)-β-CD Polymer

Figure S4. ^{1}HNMR spectrum of compound 1 Orn (Boc)-NCA in DMSO-d6

Figure S5. ^{1}HNMR spectrum of compound 2 Lys (Cbz)-NCA in DMSO-d6

Figure S6. ^{1}HNMR spectrum of compound 3PEG-Orn (Boc)-Lys (Cbz) polymer in DMSO-d6

Figure S7. ^{1}HNMR spectra of compound 4 PEG-Orn-Lys (Cbz) polymer in DMSO-d6

Figure S8. ^{1}HNMR spectra of compound 5 PEG-Orn-Lys polymer in DMSO-d6

Figure S9. ^{1}HNMR spectra of compound 6 COOH-β- CD in DMSO-d6

Figure S10. FTIR spectra of compound 6 COOH-β- CD

Figure S11. ^{1}HNMR spectra of compound 7 pALCD in DMSO-d6

Figure S12. ^{1}HNMR spectra of compound 8 N_3-pALCD in DMSO-d6

Figure S13. ^{1}HNMR spectra of compound 11 ApoE-pALCD in DMSO-d6

Figure S14. Flow cytometry analysis and semi-quantitative results validating the cholesterol clearance capacity of brush-like poly(β-CD) polymers

Figure S15. Results of the critical aggregation concentration (CAC) of brush-like poly(β-CD) polymers for cholesterol removal

Figure S16. Schematic representation of the compression and adsorption of supramolecular nanoscavengers and agarose gel electrophoresis of shSR plasmid

Figure S17. *In vitro* release of shSR plasmid from the supramolecular nanoscavengers

Figure S18. Stability assessment of supramolecular nanoscavengers

Figure S19. *In vitro* blood safety evaluation of supramolecular nanoscavengers.

Figure S20. Size and Zeta Potential of encapsulated DID fluorescent probe Supramolecular nanoscavengers

Figure S21. Results of the investigation into the modification ratio of ApoE targeting functional elements in supramolecular nanoscavengers

Figure S22. Semi-quantitative results of supramolecular nanoscavenger uptake pathways

Figure S23. Intracellular Fate of Supramolecular Nanoscavengers

Figure S24. Semi-quantitative results of supramolecular nanoscavengers *in vivo* targeted *ex vivo* tissue distribution

Figure S25. Experimental results of animal efficacy and mechanisms of supramolecular nanoscavengers

Figure S26. Flow cytometry gating strategy and complete data for tumor tissue CTL analysis

Figure S27. Flow cytometry gating strategy and complete data for the analysis of tumor-infiltrating regulatory T cell (Treg) populations in tumor tissues

Figure S28. Flow cytometry gating strategy and complete data for tumor-associated macrophage analysis in the tumor microenvironment

Figure S29. Flow cytometry gating strategy and complete data for DC cells in the cervical lymph nodes

Figure S30. Flow cytometric semi-quantification of CTL cells in splenic tissue, assessing the remodeling of the immune microenvironment at the animal level by supramolecular nanoscavengers

Figure S31. Flow cytometry gating strategy and complete data for cytotoxic T lymphocyte (CTL) cell analysis in splenic tissue, assessing the remodeling of the immune microenvironment at the animal level by supramolecular nanoscavengers

Figure S32. Supramolecular nanoscavengers animal model safety evaluation - H&E Staining Results of Heart, Liver, Spleen, Lung, and Kidney Tissues

Figure S33. Assessment of supramolecular nanoscavengers safety at the animal level results of tissue safety and blood biochemical index detection

Figure S34. Assessment of β-CD in supramolecular nanoscavengers' safety at normal brain tissues.

Table. S1 Binding energy of cholesterol/avasimibe with the host macromolecule β-CD

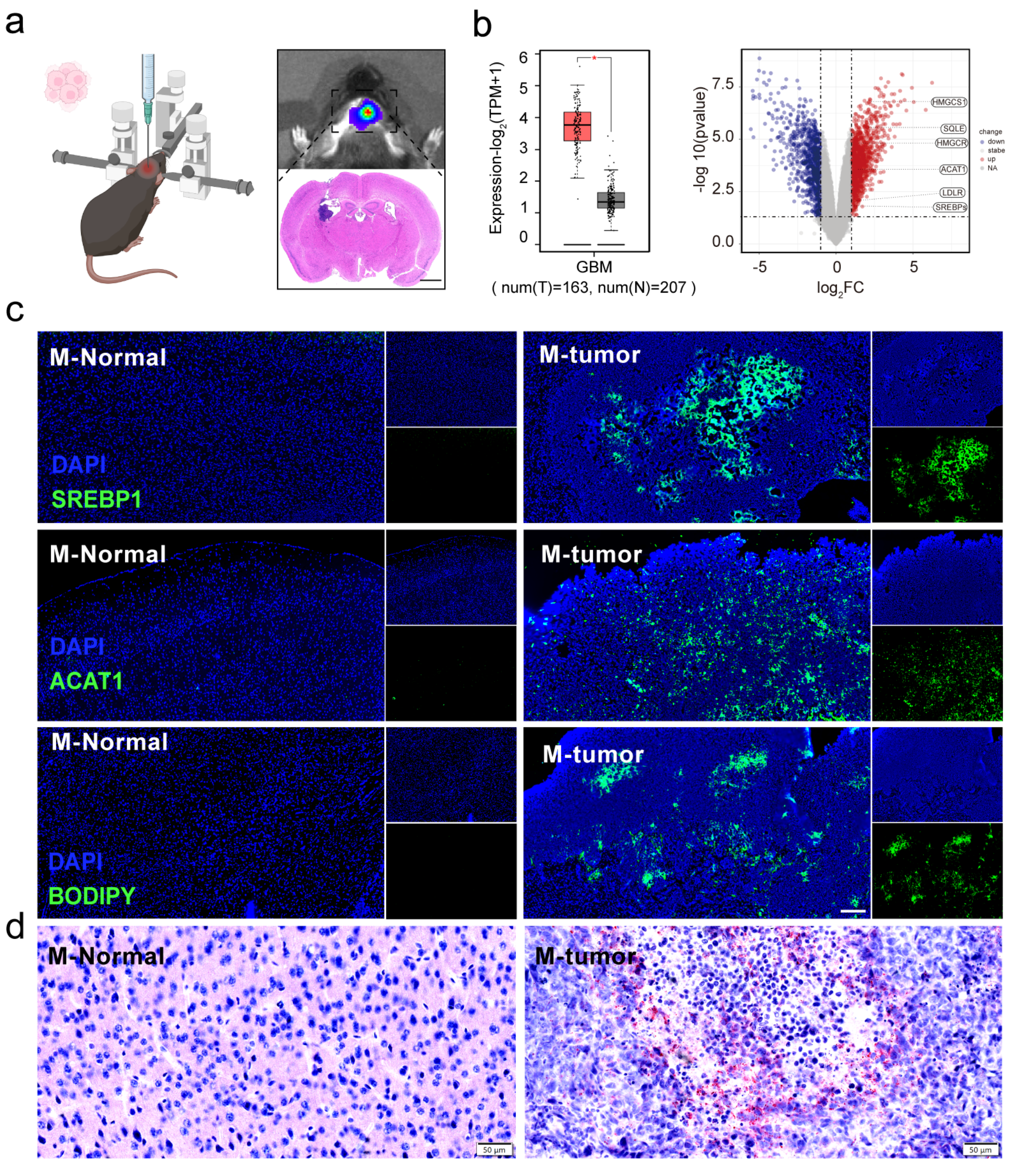

Figure S1. Validation of cholesterol reprogramming characteristics in murine GBM model. a) A schematic representation illustrating the development of the orthotopic GBM model in mouse. b) an analysis of ACAT1 expression alongside key cholesterol metabolism-related gene expression in GBM utilizing the GIPEA2 database. c) immunofluorescence findings depicting the expression of SREBP1 protein, ACAT1 protein, and free cholesterol in GBM tissues compared to normal tissues. The green color indicates SREBP1, ACAT1, or BODIPY, while blue denotes the nucleus. Scale bar measures 200 μm. d) Results from Oil Red O staining demonstrating the distribution of lipid droplets in both normal and tumor tissues of mouse. Orange or red indicates the presence of lipid droplets, with blue representing the nucleus, scale bar 50 μm.

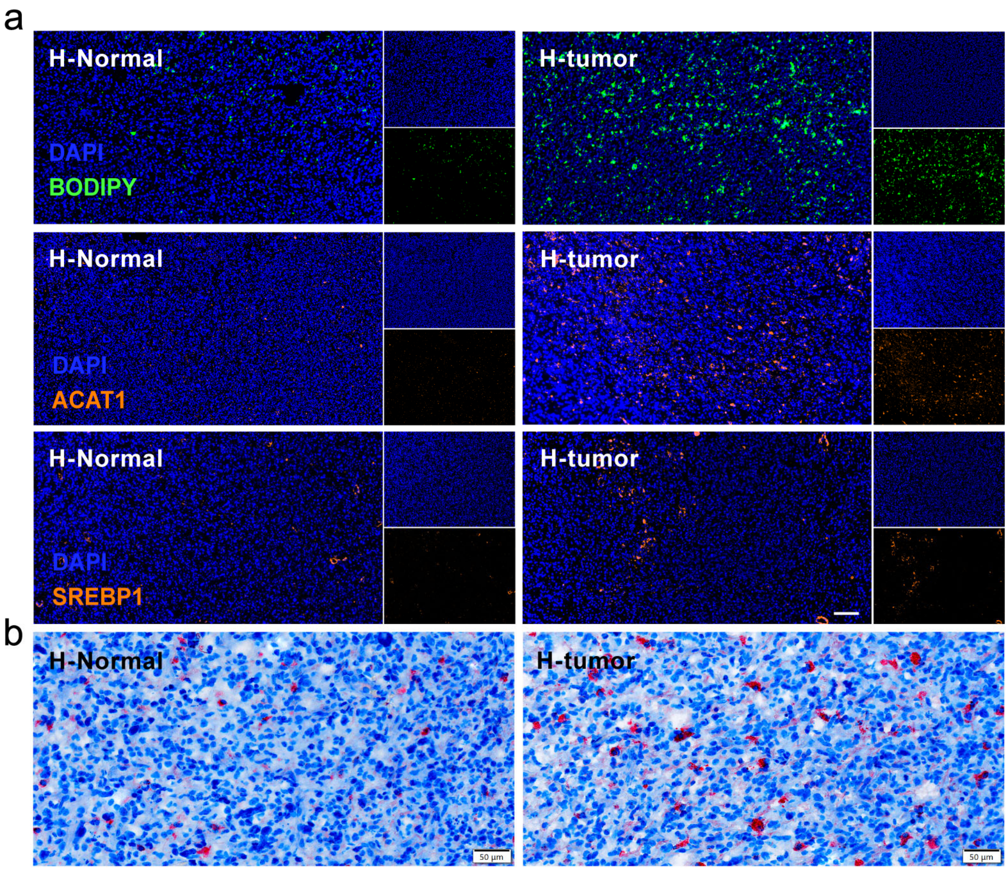

Figure S2. Validation of cholesterol reprogramming characteristics in human GBM sample. a) Immunofluorescence results shown the expression of free cholesterol, cholesterol esterification enzyme ACAT1, and SREBP1 protein in GBM tissues and normal tissues. Green represents BODIPY, orange represents ACAT1 and SREBP1, and blue represents the nucleus. Scale bar, 200 μm. b) Oil Red O staining results showing the distribution of lipid droplets in human normal and tumor tissues. Orange or red represents lipid droplets, and blue represents the nucleus. Scale bar, 50μm.

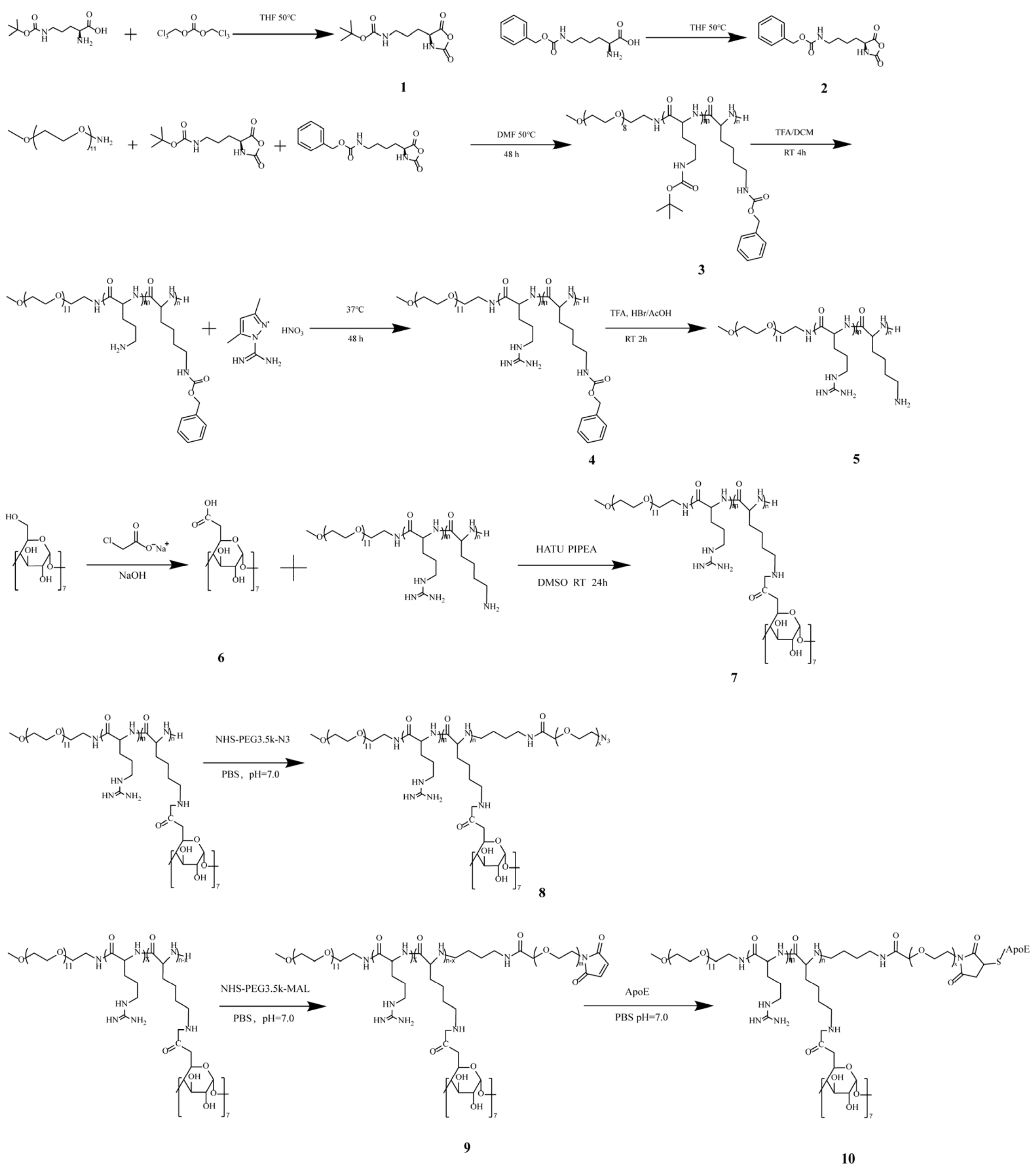

Figure S3. Synthetic Route for Brush-like Poly (amino acid)-β-CD Polymer

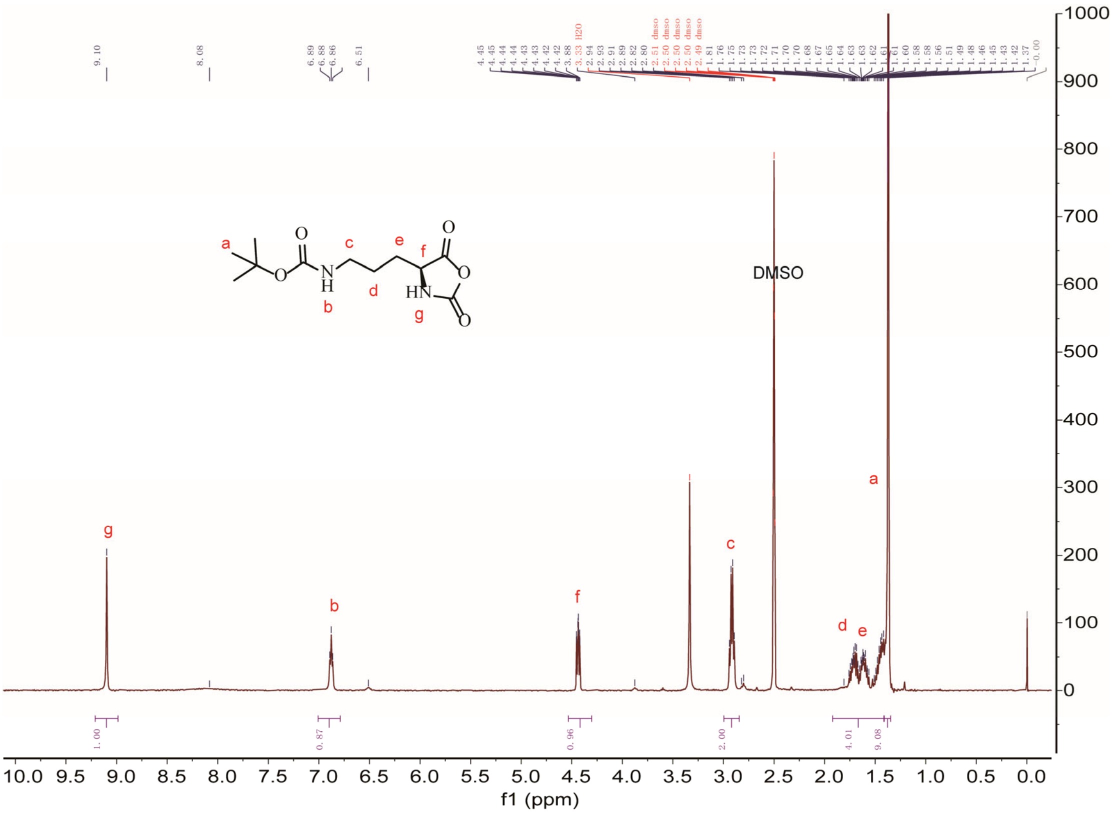

Figure S4. ^{1}HNMR spectrum of compound 1 Orn (Boc)-NCA in DMSO-d6.

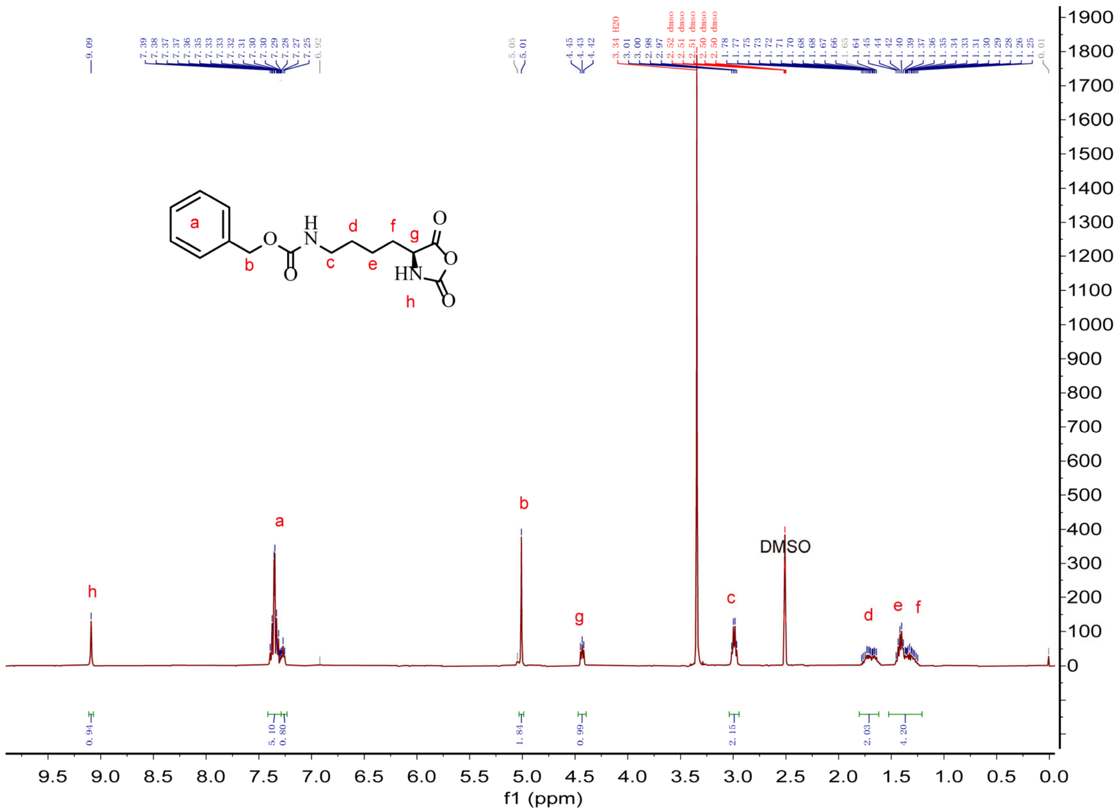

Figure S5. ^{1}HNMR spectrum of compound 2 Lys (Cbz)-NCA in DMSO-d6.

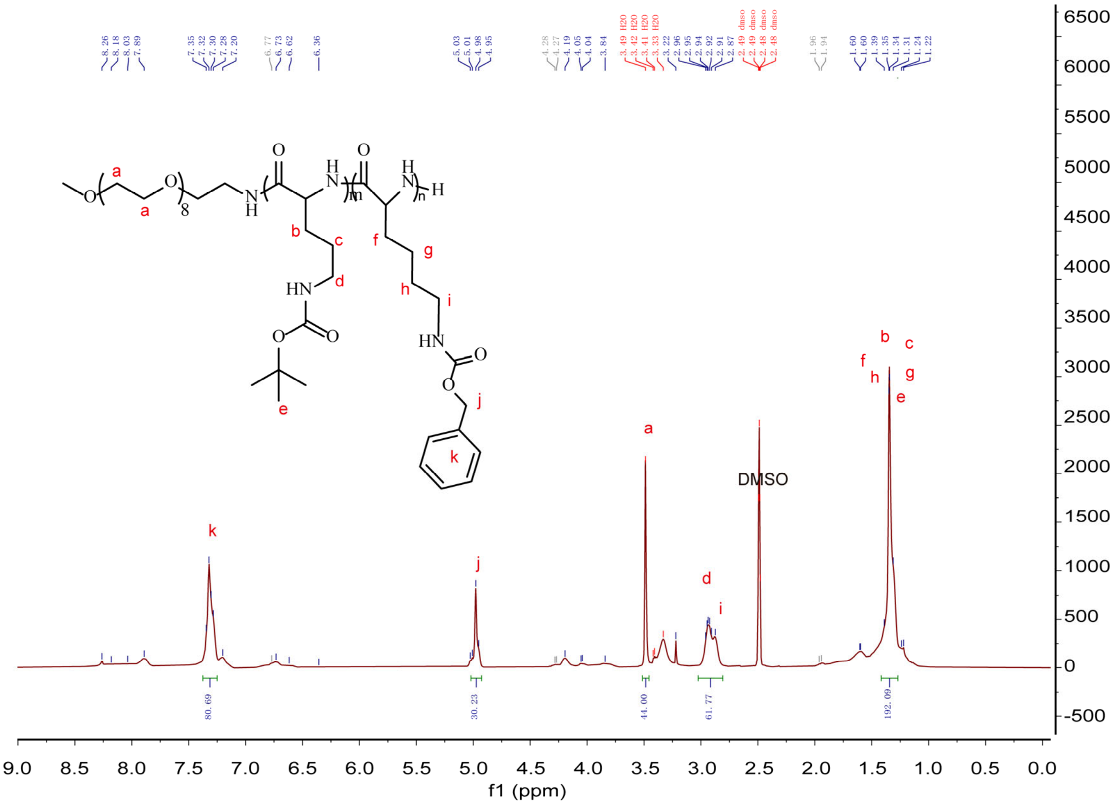

Figure S6. ^{1}HNMR spectrum of compound 3PEG-Orn (Boc)-Lys (Cbz) polymer in DMSO-d6.

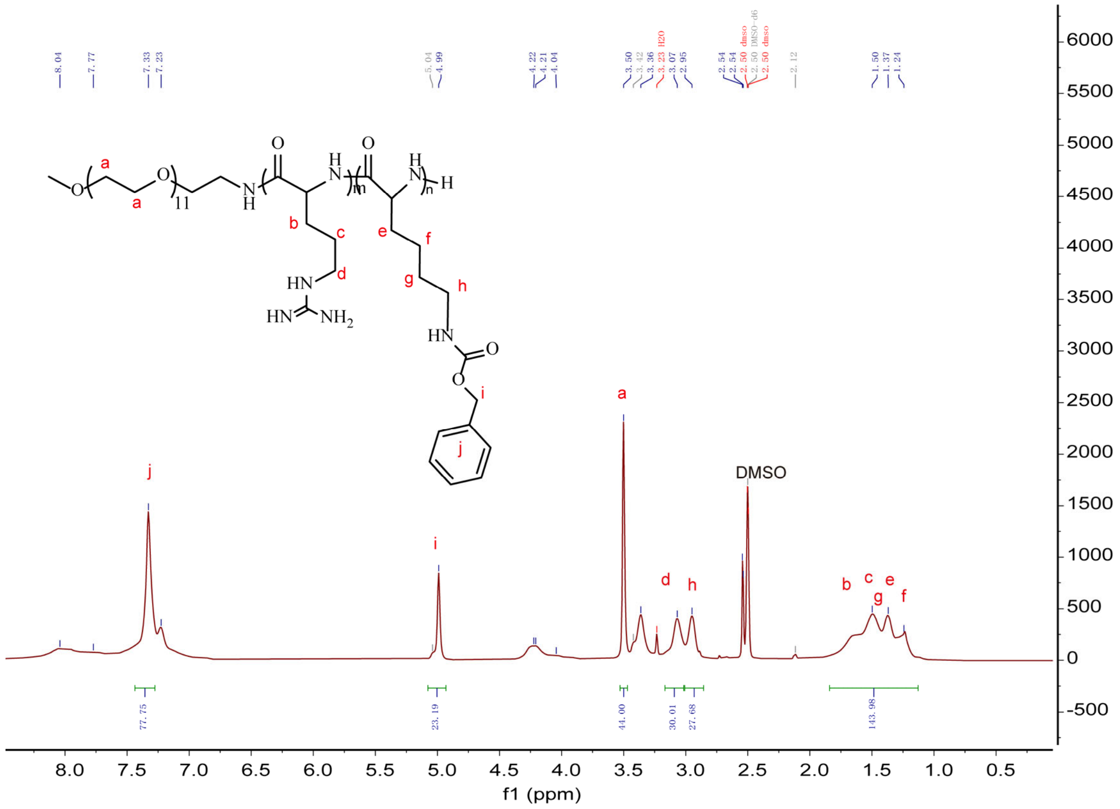

Figure S7. ^{1}HNMR spectra of compound 4 PEG-Arg-Lys (Cbz) polymer in DMSO-d6.

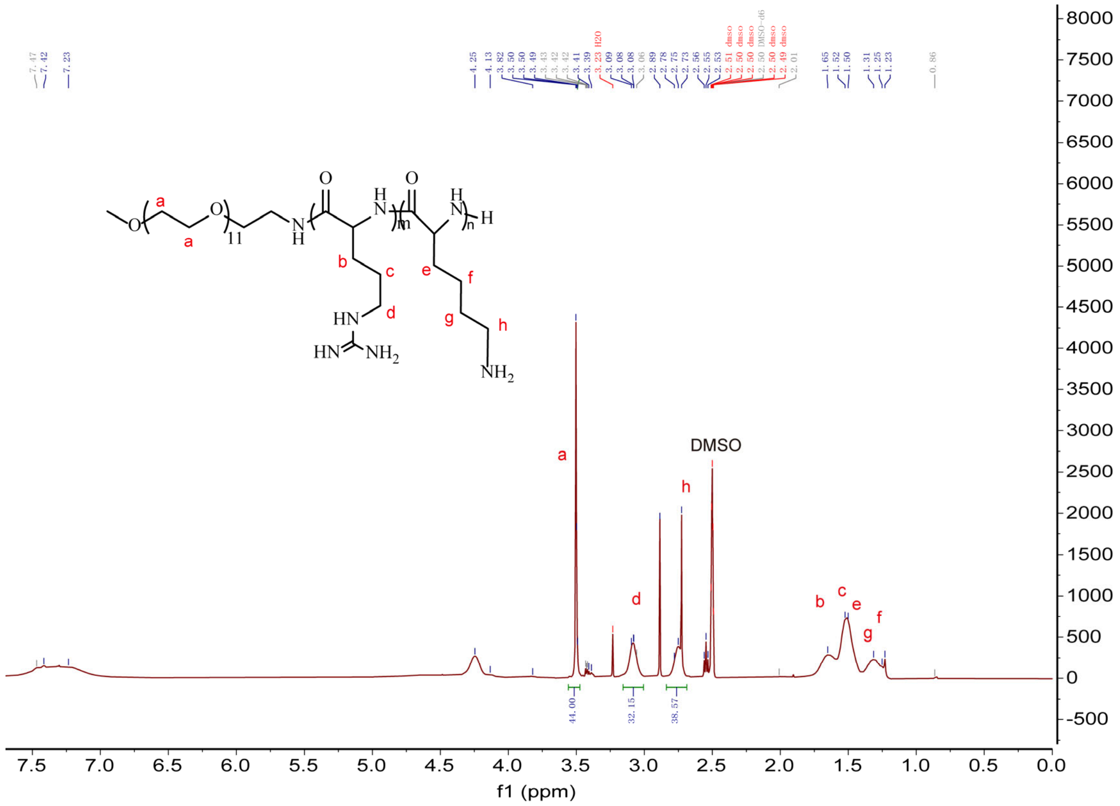

Figure S8. ^{1}HNMR spectra of compound 5 PEG-Arg-Lys (NH_2) polymer in DMSO-d6.

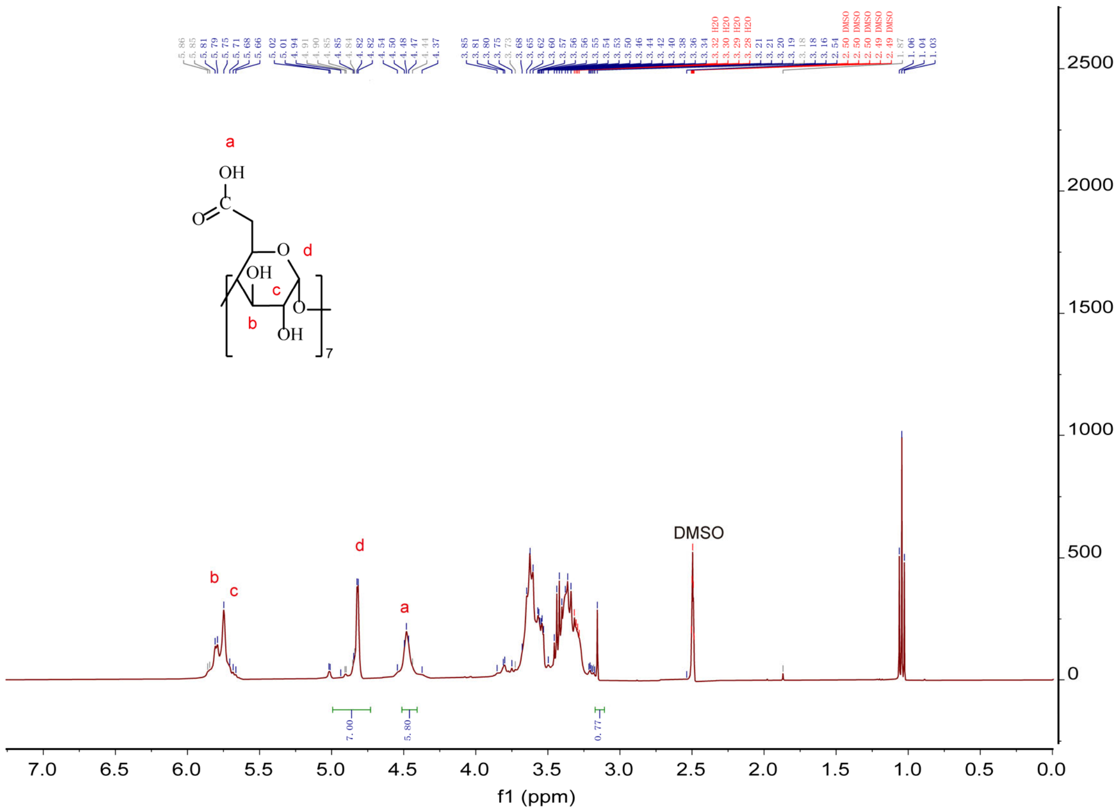

Figure S9. ^{1}HNMR spectra of compound 6 COOH-β- CD in DMSO-d6.

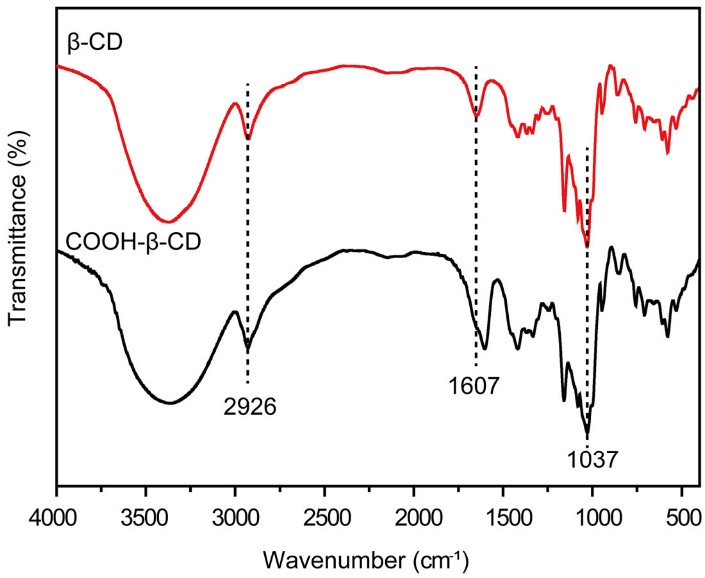

Figure S10. FTIR spectra of compound 6 COOH-β- CD.

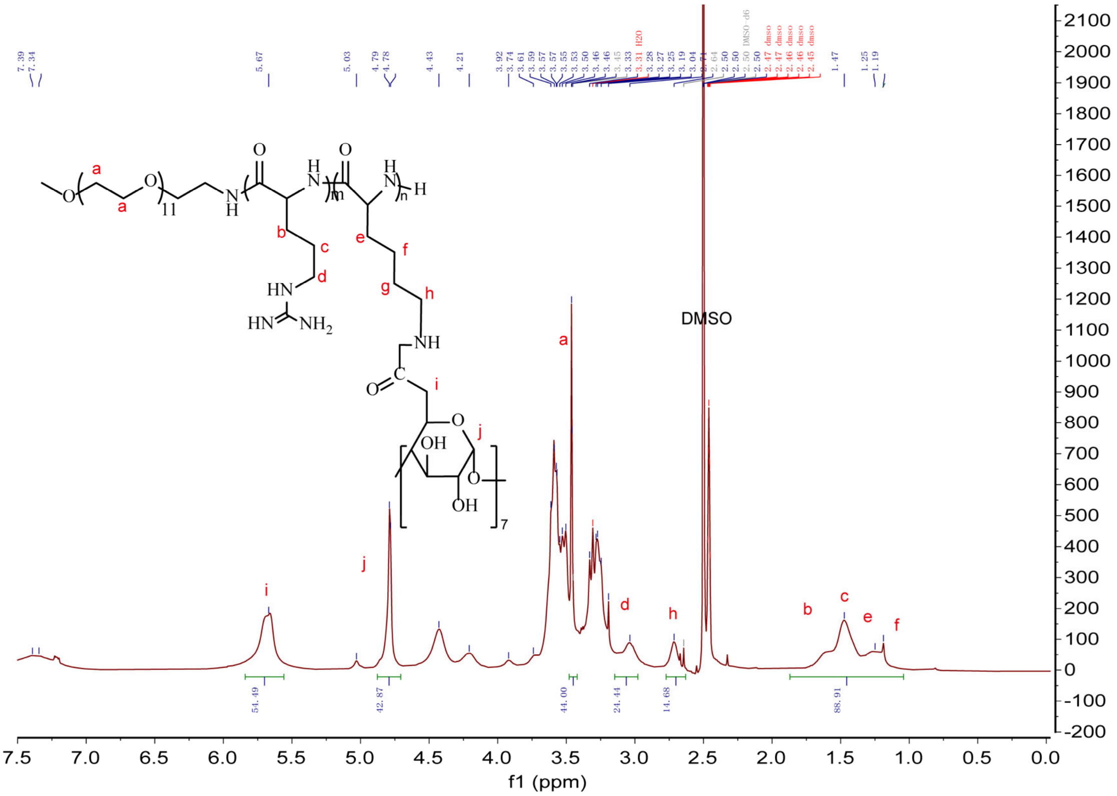

Figure S11. ^{1}HNMR spectra of compound 7 pALCD in DMSO-d6.

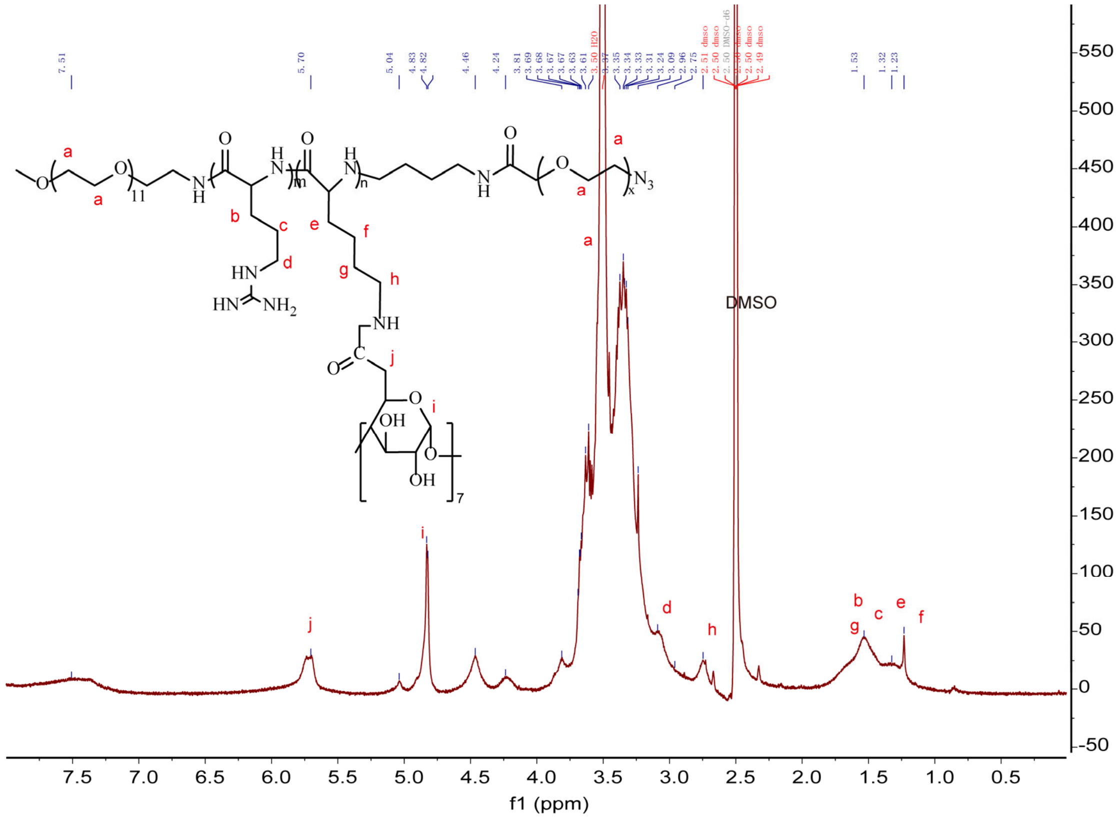

Figure S12. ^{1}HNMR spectra of compound 8 N_3-pALCD in DMSO-d6.

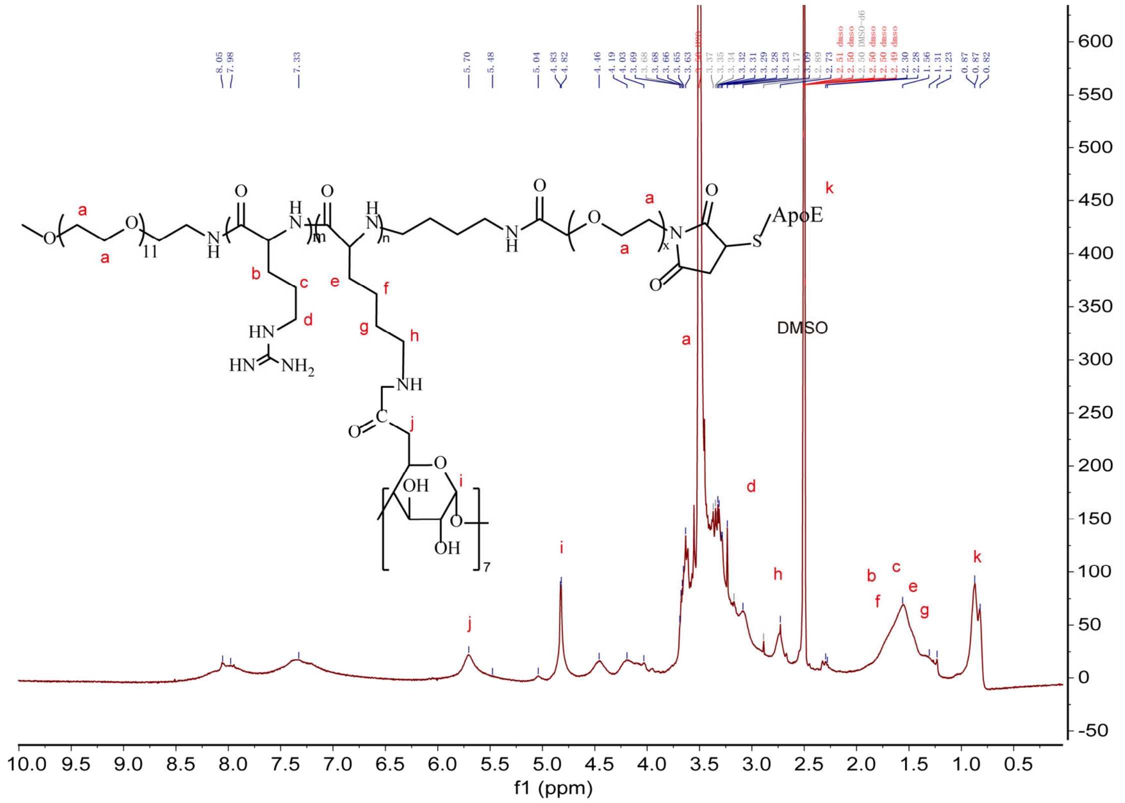

Figure S13. ^{1}HNMR spectra of compound 11 ApoE-pALCD in DMSO-d6.

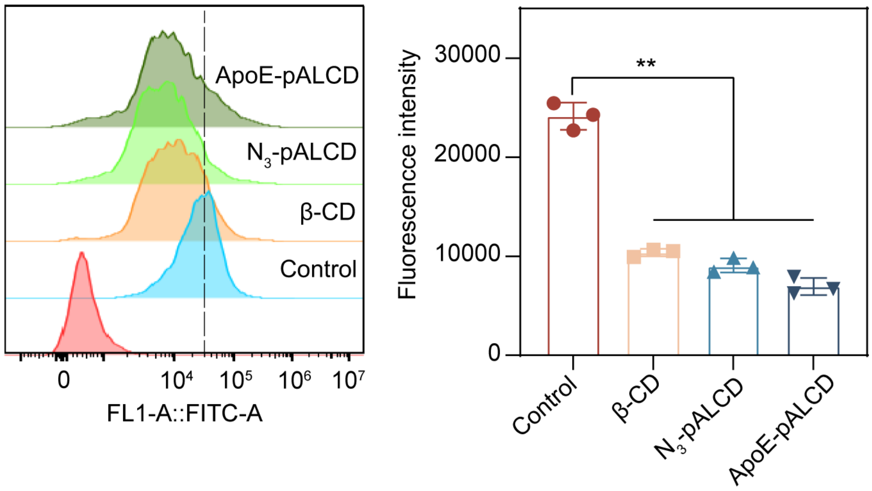

Figure S14. Flow cytometry analysis and semi-quantitative results validating the cholesterol clearance capacity of brush-like poly(β-CD) polymers. The data were shown in analytic plots as mean ± s.d., and ordinary one-way ANOVA was used. Significant differences were indicated by *$p < 0.05$*, **$p < 0.01$*, ***$p < 0.001$ *and* ****$p < 0.0001$.

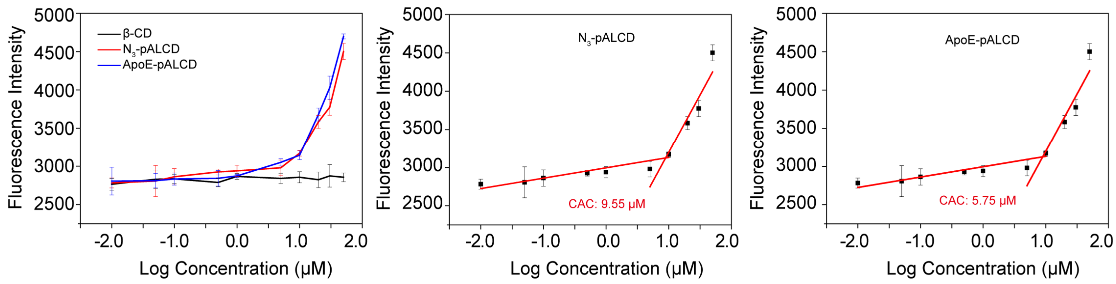

Figure S15. Results of the critical aggregation concentration (CAC) of brush-like poly(β-CD) polymers, the data were shown in analytic plots as mean ± s.d., n=3.

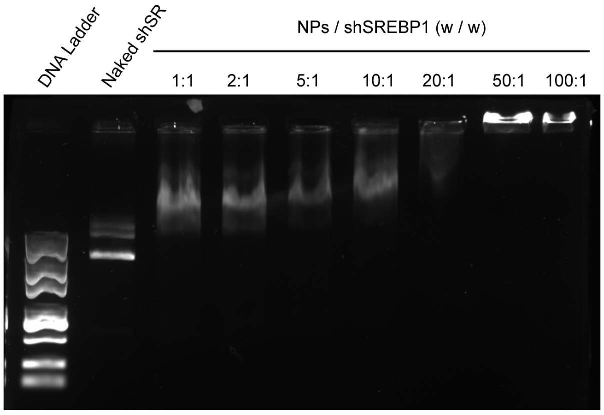

Figure S16. Schematic representation of the compression and adsorption of supramolecular nanoscavengers and agarose gel electrophoresis of shSREBP1 plasmid.

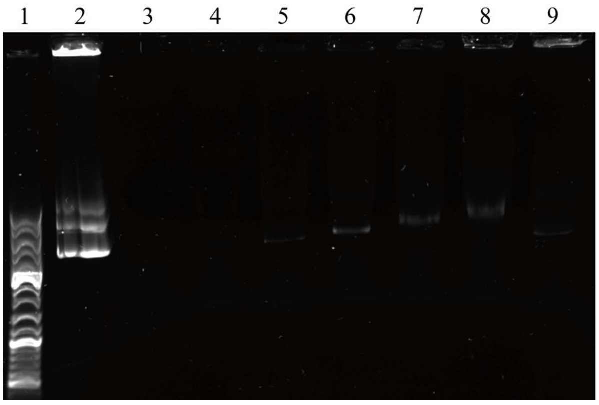

Lane 1: DNA Ladder (0.1-10 kb);
Lane 2: naked shSR;
Lane 3: released 1h;
Lane 4: released 2h;
Lane 5: released 4h;
Lane 6: released 8h;
Lane 7: released 12h;
Lane 8: released 24h;
Lane 9: released 48h;

Figure S17. *In vitro* release of shSREBP1 from the supramolecular nanoscavengers.

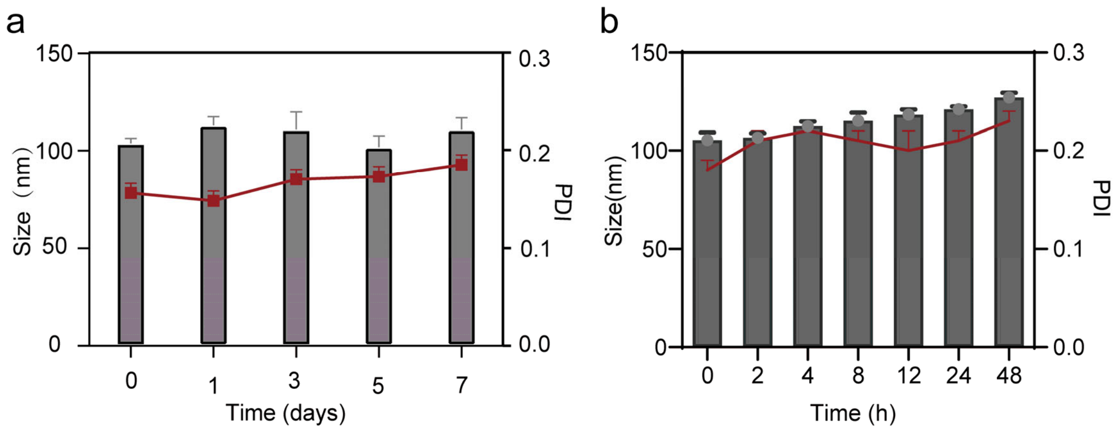

Figure S18. Stability assessment of supramolecular nanoscavengers. a) Storage stability and b) Stability under physiological conditions. The data were shown in analytic plots as mean ± s.d., n=3.

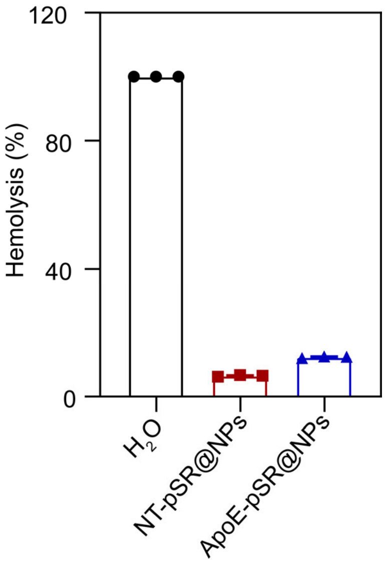

Figure S19. *In Vitro* Blood Safety Evaluation of Supramolecular nanoscavengers. The data were shown in analytic plots as mean ± s.d., n=3.

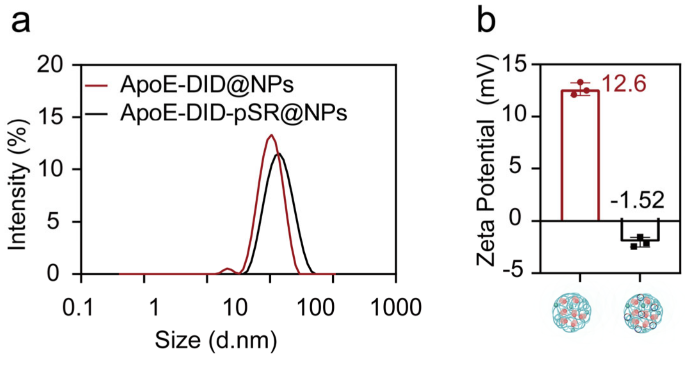

Figure S20. a) Size and b) Zeta Potential of encapsulated DID fluorescent probe Supramolecular nanoscavengers.

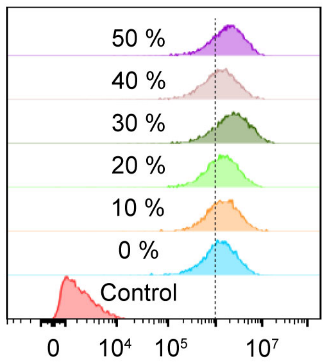

Figure S21. Results of the investigation into the modification ratio of ApoE targeting functional elements in supramolecular nanoscavengers

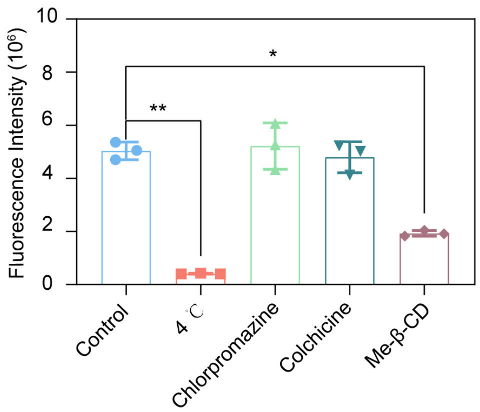

Figure S22. Semi-quantitative results of supramolecular nanoscavengers uptake pathways. The data were shown in analytic plots as mean ± s.d., and ordinary one-way ANOVA was used. Significant differences were indicated by *$p < 0.05$, **$p < 0.01$, ***$p < 0.001$ *and* ****$p < 0.0001$ (n=3).

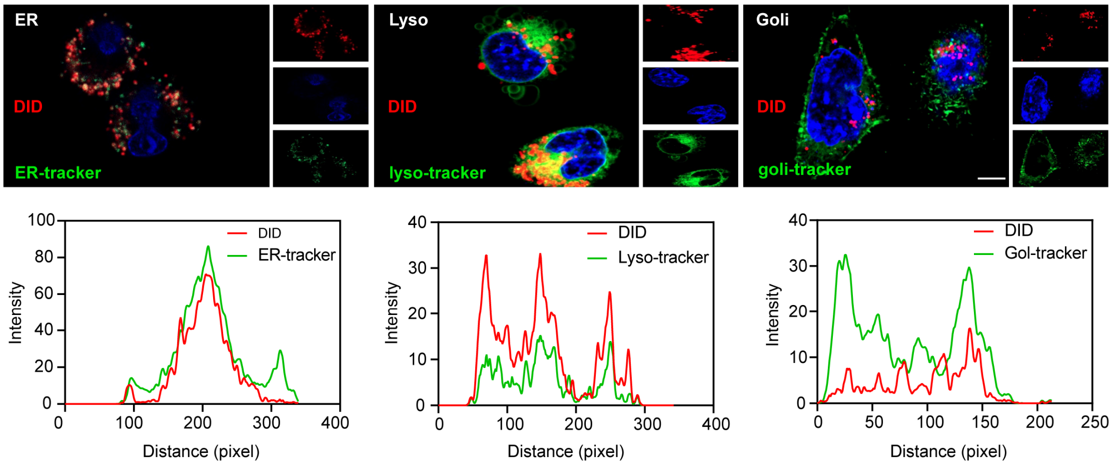

Figure S23. Intracellular Fate of Supramolecular Nanoscavengers, scale bars 20µm.

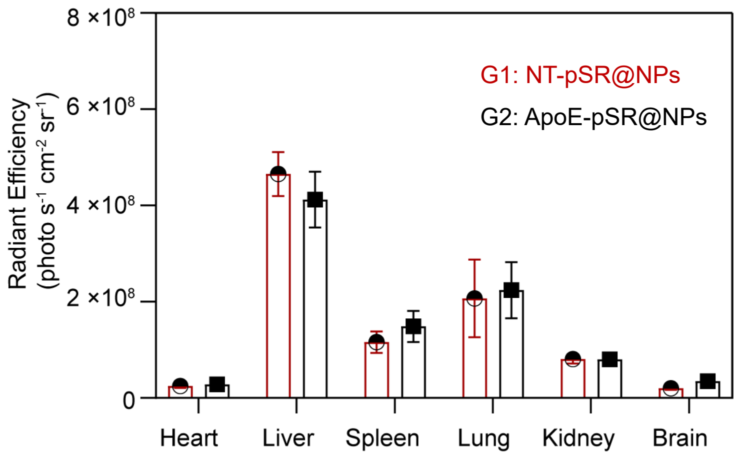

Figure S24. Semi-quantitative results of supramolecular nanoscavenger *in vivo* targeted *ex vivo* tissue distribution. The data were shown in analytic plots as mean ± s.d., n=3.

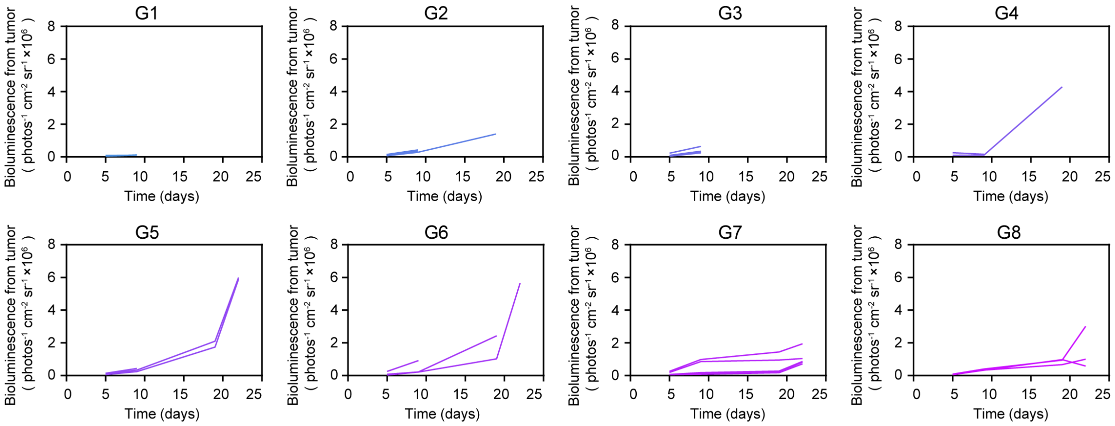

Figure S25. Experimental results of animal efficacy and mechanisms of supramolecular nanoscavengers. Tumor luciferase signals (n=4), and body weight of each group (n=4).

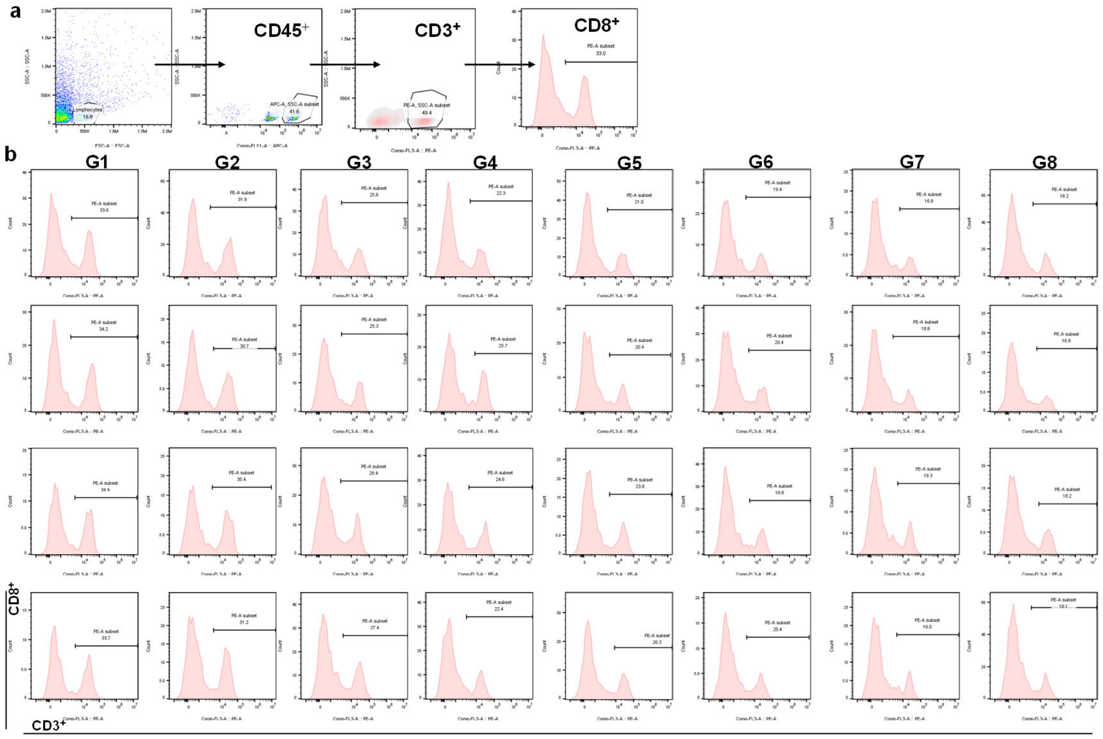

Figure S26. a) Flow cytometry gating strategy and b) complete data for tumor tissue CTL analysis, evaluating the remodeling of the immune microenvironment at the animal level by supramolecular nanoscavengers, n=4.

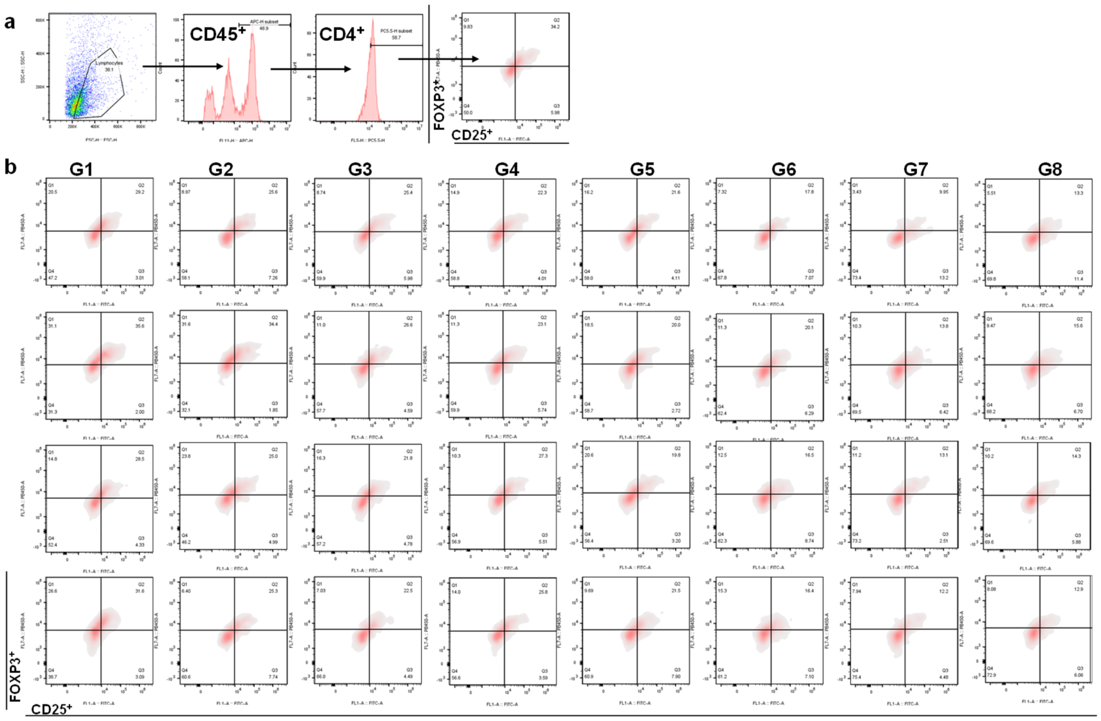

Figure S27. a) Flow cytometry gating strategy and b) complete data for the analysis of tumor-infiltrating regulatory T cell (Treg) populations in tumor tissues following supramolecular nano-scavenger treatment at the animal level, n=4.

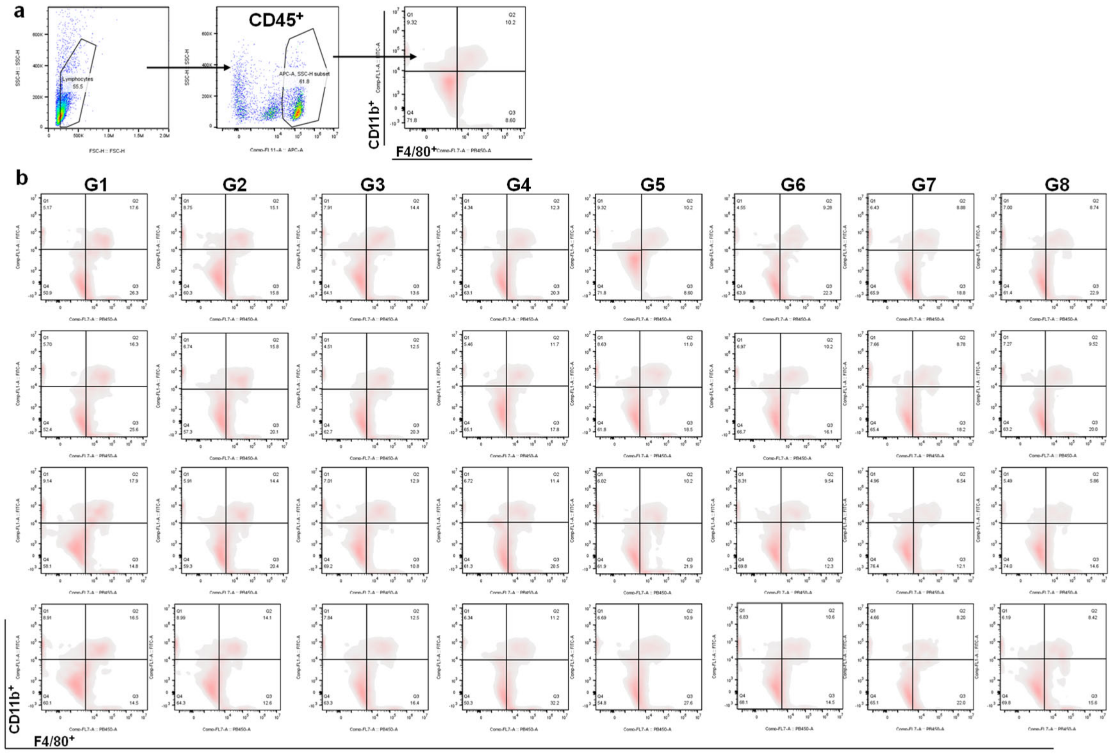

Figure S28. a) Flow cytometry gating strategy and b) complete data for tumor-associated macrophage analysis in the tumor microenvironment remodeling study at the animal level using supramolecular nanoscavengers, n=4.

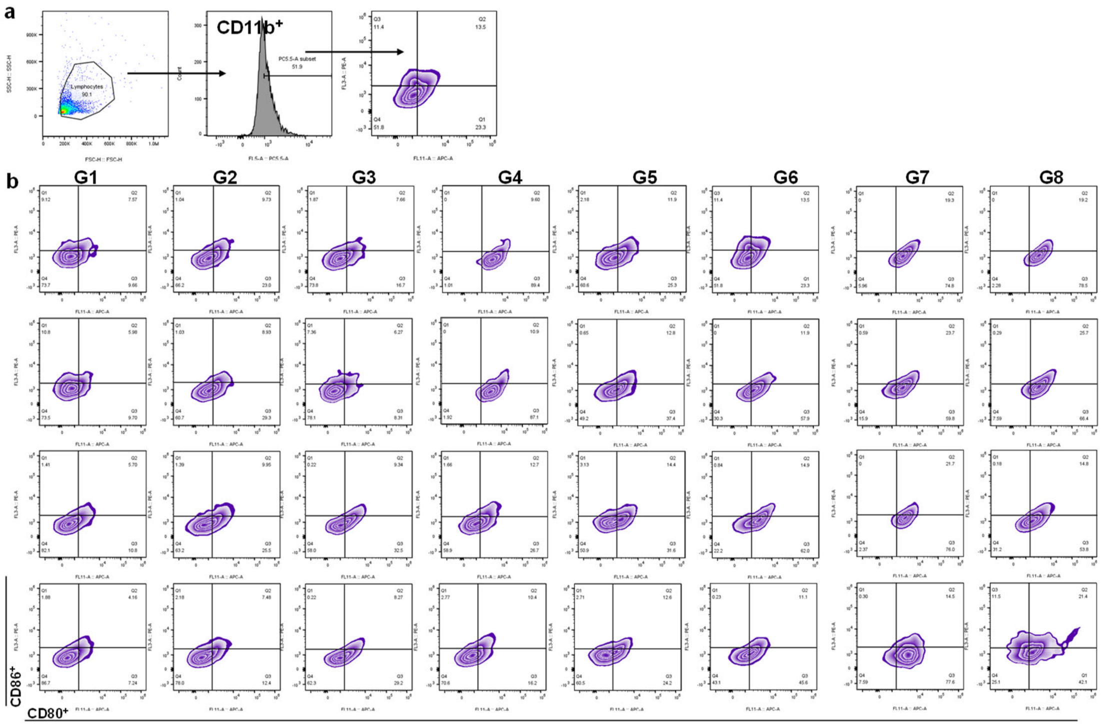

Figure S29. a) Flow cytometry gating strategy and b) complete data for DC cells in the cervical lymph nodes, assessing the remodeling of the immune microenvironment at the animal level by supramolecular nanoscavengers, n=4

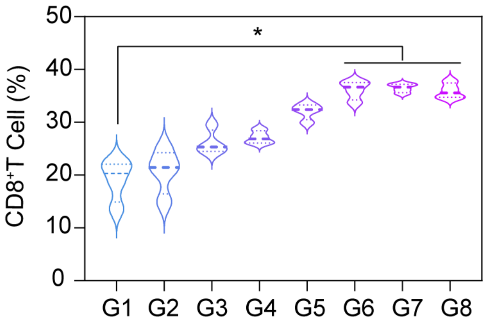

Figure S30. Flow cytometric semi-quantification of CTL cells in splenic tissue, assessing the remodeling of the immune microenvironment at the animal level by supramolecular nanoscavengers. The data were shown in analytic plots as mean ± s.d., and ordinary one-way ANOVA was used. Significant differences were indicated by $*p < 0.05$, $**p < 0.01$, $***p < 0.001$ *and* $****p < 0.0001$(n=4).

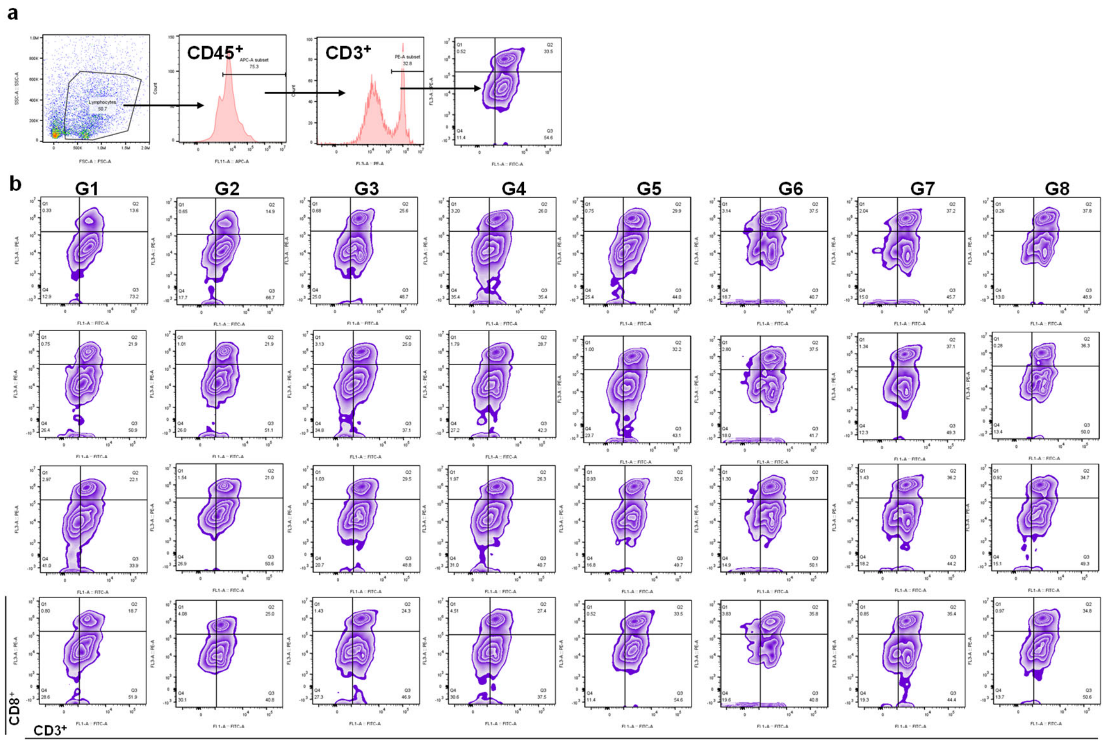

Figure S31. a) Flow cytometry gating strategy and b) complete data for cytotoxic T lymphocyte (CTL) cell analysis in splenic tissue, assessing the remodeling of the immune microenvironment at the animal level by supramolecular nanoscavengers, n=4.

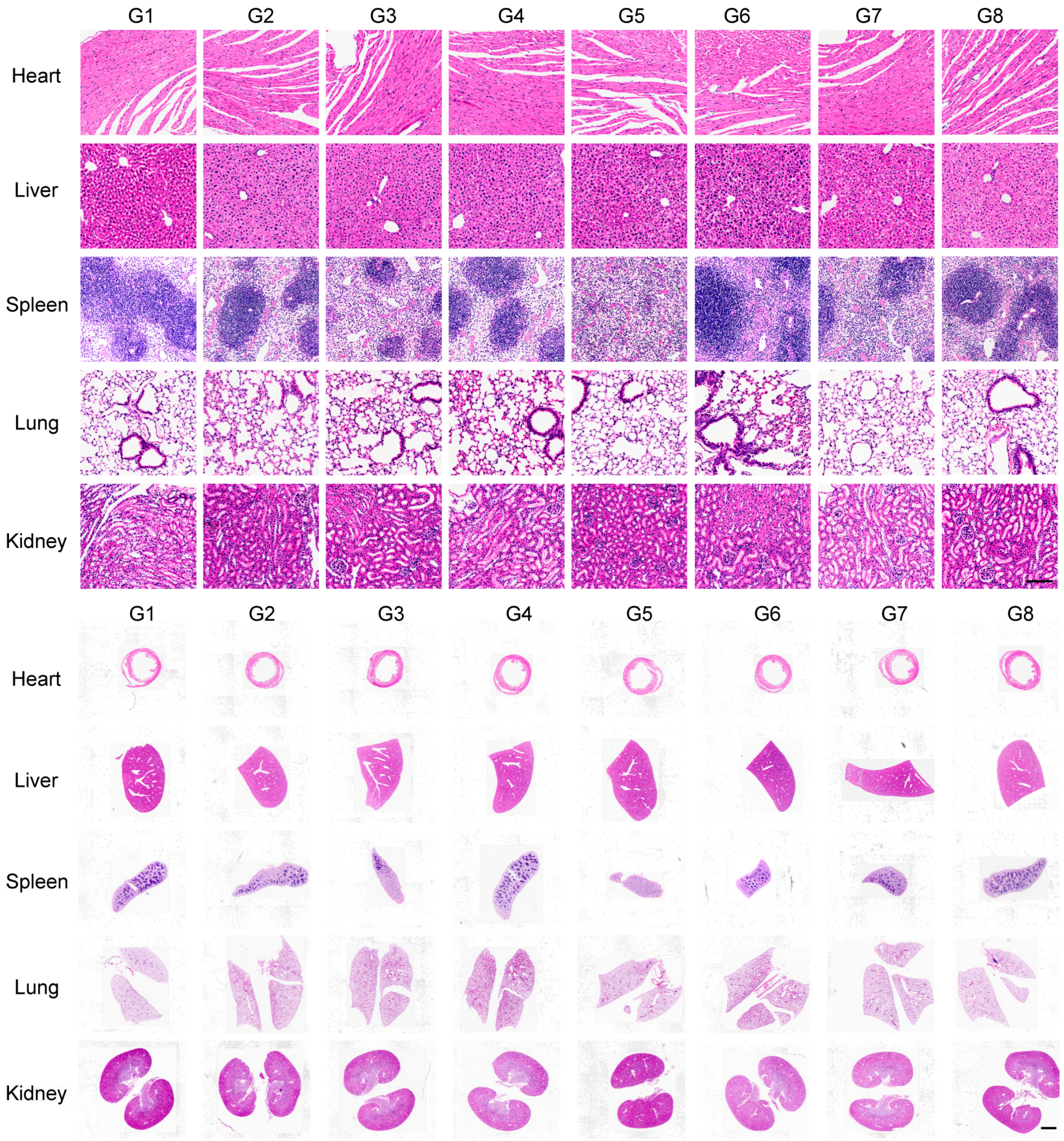

Figure S32. Supramolecular nanoscavengers animal model safety evaluation - H&E Staining Results of Heart, Liver, Spleen, Lung, and Kidney Tissues.Scale bars, 200μm and 1mm.

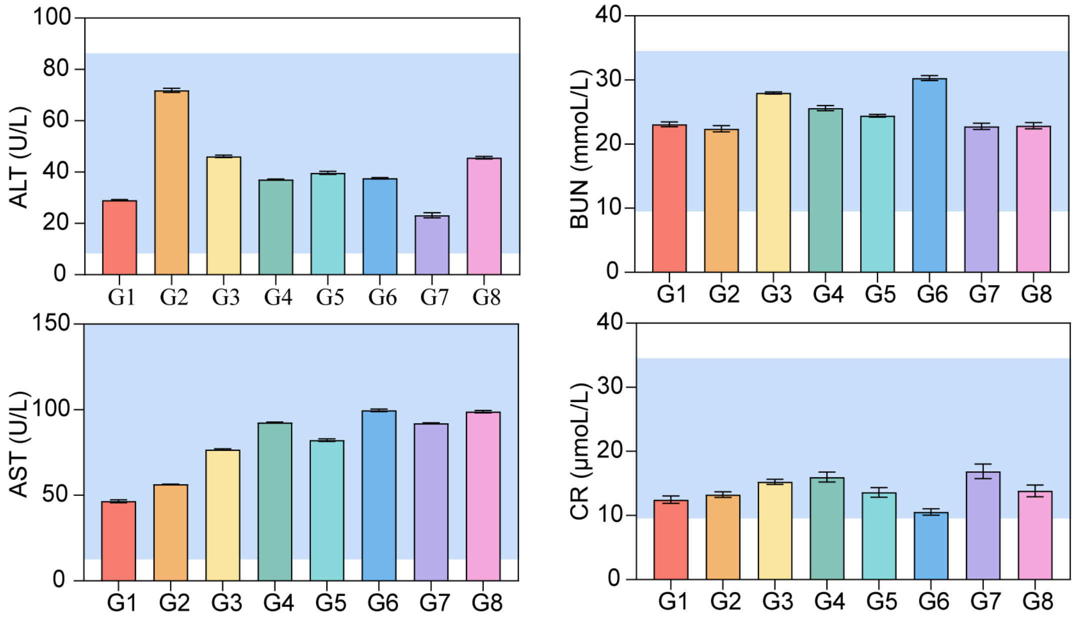

Figure S33. Assessment of supramolecular nanoscavengers safety at the animal level results of tissue safety and blood biochemical index detection, n=4.

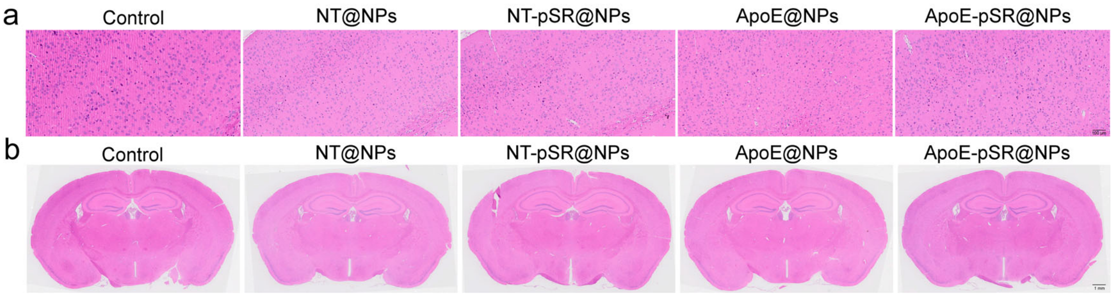

Figure S34. Supramolecular nanoscavengers animal model safety evaluation - H&E Staining Results of healthy mice brain tissues. a) detail enlargement drawing, scale bars 100 μm. b) complete brain tissue map, scale bars 1mm.

Table S1. Binding energy of cholesterol/avasimibe with the host macromolecule β-CD

conformation	Ava-β-CD Binding-energy (kcal/mol)	Cho-β-CD Binding-energy (kcal/mol)
1	-6.13	-7.29
2	-5.54	-7.18
3	-5.53	-7.17
4	-5.53	-7.17
5	-5.52	-7.16
